# Autoprobiotic Supplements Attenuate Obesity and Improve Gut Microbiota, Carbohydrate, and Lipid Metabolism in Patients with Metabolic Syndrome: A Pilot Trial

**DOI:** 10.3390/nu18142324

**Published:** 2026-07-16

**Authors:** Elena Ermolenko, Stanislav Sitkin, Lyubov Alferova, Nadezhda Novikova, Nikita Gladyshev, Victoria Orlova, Alena Zalicheva, Elena Demchenko, Oleg Ergashev, Alexander Suvorov

**Affiliations:** 1 Institute of Experimental Medicine, St. Petersburg 197376, Russianadezhda.lavrenova.vrn@gmail.com (N.N.); alexander_suvorov1@hotmail.com (A.S.); 2 Department of Medical Microbiology, North-Western State Medical University Named After I.I. Mechnikov, St. Petersburg 191015, Russia; 3 Department of Internal Diseases, Gastroenterology and Dietetics Named After S.M. Ryss, North-Western State Medical University Named After I.I. Mechnikov, St. Petersburg 191015, Russia; 4 Almazov National Medical Research Centre, St. Petersburg 197341, Russia; 5 Petrovsky National Research Centre of Surgery, Moscow 117418, Russia

**Keywords:** autoprobiotic, metabolic syndrome, obesity, lipid metabolism, fecal microbiota, *Enterococcus* spp., *Bacteroides fragilis* group, *Senegalimassilia*, *Oscillospiraceae UCG-003*, glucose

## Abstract

**Background/Objectives**: A pilot study was conducted to evaluate the effectiveness of treatment with autoprobiotic bacteria from indigenous, non-pathogenic *Enterococcus faecium* and *Enterococcus hirae* strains in patients with metabolic syndrome (MetS). **Methods**: Fifty patients with MetS (sex-matched, aged 42–63 years) were randomized to an experimental group (Ap, n = 26) that received autoprobiotics grown in nutritional mix SuproPlus 2640 and a control group (Pl, n = 24) that received SuproPlus 2640 for 20 days. **Results**: The effects of therapy on anthropometric and biochemical parameters, as well as the gut microbiome, were assessed on days 14 and 28 after the autoprobiotic course. Autoprobiotic treatment reduced the severity of obesity symptoms and led to decreases in serum glucose and glycated hemoglobin (HbAc1) levels and partial normalization of the lipid profile. An intergroup comparison revealed lower concentrations of HbAc1 and triglycerides in blood serum when comparing samples taken from Ap and Pl groups on day 28 after therapy. qPCR showed a reduction in the numbers of *Bacteroides fragilis* group, *Streptococcus* spp., and *Ruminococcus* spp. in the Ap group. The 16S rRNA gene sequencing results provided a longitudinal model for relative abundance analysis of the observed taxa, and comparison between dynamic parameters indicated a more favorable trend in the Ap group, with a decrease in the relative abundance of *Oscillospiraceae UCG-003* and an increase in that of “*Prevotellamassilia*” observed only in this group. Longitudinal microbiota analysis using the coda4microbiome package demonstrated that the most pronounced microbiome shifts occurred in the Ap group, with the genera *Senegalimassilia*, “*Prevotellamassilia*”, *Streptococcus*, *Paraprevotella*, and *Anaerobutyricum* contributing substantially. **Conclusions**: Autoprobiotic *Enterococcus* spp. may affect the gut microbiome and is potentially effective for treating MetS.

## 1. Introduction

Metabolic syndrome (MetS), characterized by visceral obesity, dyslipidemia, hyperglycemia, and hypertension, has emerged as a significant global public health challenge [1,2,3,4,5]. Over the past decade, advances in microbiome research have highlighted that many risk factors for MetS may be primarily associated with the diversity, composition, and function of the gut microbiota [6,7,8,9,10,11,12,13,14,15,16,17,18].

While studies investigating the characteristics of the intestinal microbiota in patients with obesity, diabetes mellitus, cardiovascular disease, atherosclerosis, and non-alcoholic fatty liver disease (NAFLD) show considerable variability, common trends are consistently reported. These include reduced microbial diversity [19]; a decline in the abundance of lactobacilli, bifidobacteria, eubacteria, and ruminococcin; and an increase in the abundance of opportunistic bacteria, particularly staphylococci, streptococci, and members of the *Enterobacteriaceae* family. This shift is associated with low-grade inflammation, characterized by elevated blood concentrations of lipopolysaccharides [20,21,22,23,24,25]. Studies suggest that the gut microbiota in individuals with MetS may extract dietary energy more efficiently, potentially leading to higher body mass index (BMI) and insulin resistance [26].

In addition, researchers have focused on microbial producers of short-chain fatty acids (SCFA); in particular, butyrate, propionate, lactate, and acetate, which have beneficial metabolic effects, stabilize intestinal functions and permeability, enhance mitochondrial activity, prevent metabolic endotoxemia, and activate gluconeogenesis and other hormonal regulation pathways. There is a scientific consensus that butyrate-producing bacteria can both contribute to and inhibit the development of obesity by modulating the composition and metabolism of the gut microbiota, thereby preventing dysbiosis and chronic inflammation that contribute to MetS [27].

In addition to diet and increased physical activity, lifestyle changes, pharmaceuticals (such as antidiabetic agents and statins), bariatric surgery, prebiotics, and probiotics were used in the treatment of MetS (particularly obesity) [28].

Correction of intestinal dysbiosis to restore metabolic homeostasis in MetS can be achieved with the application of appropriate probiotics or postbiotics, based on the strains from the genera *Bifidobacterium*, *Akkermansia*, *Bacteroides*, *Enterococcus*, and the *Lactobacillaceae* family, which helps in reducing intestinal permeability, restoring gut function, and rectifying microbiota imbalances [29,30,31]. Studies have shown that oral probiotic application/consumption can lower serum glucose levels and modulate lipid metabolism in animal models [29,31,32,33]. Moreover, some studies have explored the effects of probiotics on glucose and lipid profiles in humans, but the results have been inconsistent [34,35], likely due to variations in the probiotic strains used [36]. This topic merits further scientific attention and additional trials. Nevertheless, it has been demonstrated that probiotics can improve the prognosis of type 2 diabetes mellitus (T2DM) by modulating the gut microbiota, stimulating insulin production, enhancing tissue sensitivity to insulin [37], and reducing oxidative damage to pancreatic tissues [38]. They have also shown beneficial effects on lipid profiles [39,40]. Several possible mechanisms by which probiotics prevent, control, and combat obesity have been proposed, including enhancing beneficial microbiota, suppressing pathogens and pathobionts, preventing the penetration of pathogens into the intestinal mucosa, enhancing the mucosal barrier, producing antimicrobial compounds, regulating the immune system, reducing proinflammatory molecules, and stimulating beneficial metabolites [40].

However, based on meta-analyses, some authors argue that probiotics may not be universally superior to conventional treatments and lifestyle interventions. Their effects are strain-specific and dose-dependent, and require long-term administration (8–12 weeks) [41]. The short duration of probiotic efficacy is attributed to their rapid clearance from the host, likely due to immune responses, unsuitable microenvironments, and suboptimal growth conditions. The complexity of the intestinal microbiota must also be considered; in some cases, probiotics have been shown to be ineffective, reducing levels of beneficial indigenous microorganisms and even provoking inflammation [42].

As an alternative approach, autoprobiotics have gained interest. Autoprobiotics are prepared from beneficial, non-pathogenic, obligate members of the host’s own (indigenous) microbiota. These strains are isolated, processed into a personalized functional food product (PFFP), and reintroduced into the same individual in doses comparable to conventional probiotic regimens [39]. The specific efficacy and safety of autoprobiotics, including those derived from non-pathogenic strains of enterococci, lactobacilli, and bifidobacteria, as well as their combinations, have been demonstrated in vitro for antimicrobial activity and in vivo in experimental models of antibiotic-associated dysbiosis [39,43,44].

The authors selected *Enterococcus faecium* and *Enterococcus hirae* strains for exploration as autoprobiotics, due to their potential metabolic effects. These beneficial effects include (but are not limited to) a hypercholesterolemic effect closely related to the deconjugation of bile salts [45], an anti-atherosclerotic effect mediated by an increase in the SCFA level [46], and strengthening the intestinal epithelial barrier by improving the transport and utilization of butyrate [47], as well as anti-obesity and antidiabetic effects through modulating the gut microbiota, including by elevating the abundance of *Lactobacillales* [48]. Additionally, *Enterococcus* spp. can exert an anti-adhesive effect against pathogens [49].

Indigenous strains of lactobacilli, bifidobacteria, and enterococci are typically used as autoprobiotics in PFFPs, with *Enterococcus* strains preferred due to their ease of isolation, cultivation, and storage. Autoprobiotic *Enterococcus* strains have been extensively studied in biological models and clinical trials. They are effectively used in the Russian Federation to treat irritable bowel syndrome, colorectal cancer, Parkinson’s disease, campylobacteriosis, *Helicobacter pylori* infection, and other infectious and non-infectious diseases [39,43,50,51,52].

Given their promising results in clinical studies, the role of autoprobiotic *Enterococcus* in various pathological processes warrants further investigation. This study aimed to assess the efficacy of indigenous *Enterococcus* strains in patients with MetS by analyzing anthropometric measures, serum biochemistry, and gut microbiota.

## 2. Materials and Methods

### 2.1. Characteristics of Patients

The potential autoprobiotic enterococcal strains were isolated from 50 patients, divided into two groups, Ap (n = 26) and Pl (n = 24), who received an autoprobiotic or SuproPlus 2640, respectively (Figure S1, Supplementary Materials). An equal number of men and women were included in each group. The study groups are detailed in Table 1.

The groups did not differ in gender, age, or the presence or absence of hypertension and coronary heart disease in the anamnesis. All patients signed a voluntary informed consent to participate in the study. The study involved men and women aged 42 to 63 years. All patients were overweight or had abdominal obesity (BMI > 25 kg/m^2^ and waist circumference ≥ 94 cm in men and ≥80 cm in women). The study participants exhibited changes in their lipid profile, characterized by hyperlipidemia of types IIa and IIb according to the Fredrickson classification, as well as a disorder of carbohydrate metabolism, manifesting as impaired glucose tolerance. In the anamnesis, 69% (Ap group) and 67% (Pl group) of patients were diagnosed with hypertension, which was combined with coronary heart disease in 31% (Ap group) and 33% (Pl group). The study did not include patients with concomitant diseases that require constant or long-term therapy, the influence of which could affect the results of the study; cancer and myeloproliferative diseases; substance or alcohol abuse; in a state of pregnancy or at the stage of pregnancy planning; and taking antibacterial, antiviral, antifungal, or antiprotozoal drugs. If the patient needed to change therapy (or it was recently changed), or if there were recent lifestyle changes (nutrition, physical activity), then the patient’s inclusion was postponed until stabilization for 2–4 weeks. The conditions for inclusion in the study were stable therapy, a stable diet, and a stable level of physical activity.

### 2.2. Randomization and Blinding

Upon enrollment, eligible participants were assigned a unique identification number and subsequently randomized in a 1:1 ratio using block randomization to one of the treatment groups. Randomization was performed by an independent investigator who was not involved in participant recruitment, clinical assessments, or outcome evaluation, and who was blinded to the participants’ identities. This investigator maintained the list of unique identification numbers and the corresponding intervention assignments in a sealed envelope stored in a secure location. The investigational product and SuproPlus 2640 were identically packaged and dispensed with no indication of group allocation on the labels. Throughout the study, all investigators, study coordinators, and participants remained blinded to the group allocations.

The treatment allocation was unblinded after the completion of data collection, database lock, and final approval of the statistical analysis plan, immediately prior to conducting the final statistical analysis (Tables S1 and S2, Supplementary materials).

### 2.3. Ethical Considerations

The study received approval from the Local Ethics Committee of Almazov National Medical Research Centre (15 May 2023, ref.: 05–23), St. Petersburg, Russian Federation. This clinical study was registered on the ISRCTN (International Traditional Clinical Trials Registry) with study registration number ISRCTN30134752 (5 June 2026). All patients signed an informed consent form approved by the Local Ethics Committee, in accordance with the principles outlined in the World Medical Association Declaration of Helsinki. The study adhered to the “Rules of Clinical Practice in the Russian Federation” approved by the Ministry of Health of the Russian Federation (Order No. 266, 19 June 2003). It was authorized under Federal Law No. 323-FL dated 21 November 2011, “On Protection of Health of Citizens in the Russian Federation.” The study maintained patient confidentiality.

### 2.4. Preparation of Autoprobiotics

The methodology for producing autoprobiotics based on non-pathogenic *E. faecium* or *E. hirae*, previously developed by our team [53], was employed. Within 3 days before material collection, patients with MetS were required to discontinue use of laxatives, cleansing enemas, probiotics, antibacterial and chemotherapeutic drugs, dietary supplements, and lactic acid products. Additionally, they were asked to refrain from using psychoactive substances and alcohol. Fecal samples (0.5 g, preferably 5.0 g) were collected from patients to prepare an autoprobiotic starter culture and delivered to the laboratory within 2 h after defecation. The fecal suspension obtained after mixing 100 µg of feces with 1000 µL of phosphate-buffered saline (consisting of NaCl: 137 мM, KCl: 2.7 мM, Na_2_HPO_4_: 10.1 мM, K_2_HPO_4_: 1.38 мM, pH 7.4) was seeded on an enterococcal agar containing sodium azide and crystalline violet dye (NICF, St. Petersburg, Russian Federation) and was cultured for 48 h at a temperature of 37 °C aerobically. The plates with this selective medium predominantly supports the growth of enterococci, with occasional growth of staphylococci.

Presumptive species-level identification was performed based on distinct colony morphology. Specifically, *Enterococcus faecalis* colonies typically appeared dark burgundy, whereas *Enterococcus faecium* and *E. hirae* colonies exhibited a lilac-pink coloration with a pale margin. Pure cultures were verified via Gram staining and examined under a light microscope. Microscopically, *Enterococcus* spp. appeared as Gram-positive cocci arranged in short chains with a tendency to aggregate. The confirmed pure cultures were subsequently subjected to genetic analysis. Pure *Enterococcus* cultures were obtained after selecting three individual colonies, from each of which DNA had been isolated. *Enterococcus faecium or E. hirae* strains were identified using a polymerase chain reaction (PCR) as described earlier [54].

Additionally, we used MALDI-TOF (matrix-assisted laser desorption/ionization time-of-flight mass spectrometry; Bruker Daltonics, Bremen, Germany) for species identification of enterococci. Indigenous strains of *E. faecium* or *E. hirae* were tested by PCR with electrophoretic detection for the absence of pathogenicity genes and vancomycin resistance genes [54]. The DNA primers used to determine *Enterococcus* pathogenicity and antibiotic resistance genes are listed in Table S1. We examined several pure enterococcal cultures and selected those that met the criteria: belonging to the species *E. hirae* or *E. faecium* and not having been analyzed for pathogenic genes and antibiotic resistance. Exclusion criteria were genes providing adhesion and colonization, tissue damage, hemolysis and production of toxins, and resistance to vancomycin, erythromycin and vancomycin (Tables S3 and S4, Supplementary Materials).

Selected non-pathogenic individual enterococcal strains were used to prepare 10 mL of primary starter culture in the nutrient medium (SuproPlus 2640, 40 g/L; Monsanto Company, St. Louis, MO, USA), which was incubated for 48 h at 37 °C under aerobic conditions. Subsequently, the resulting 10 mL of starter culture was used as seed material to produce 1 L of autoprobiotic product (PFFP). The finished individual autoprobiotic contained 5 × 10^8^ colony-forming units (CFU) of the indigenous *Enterococcus* strain in 1 mL and was recommended for administration in 50 mL twice daily by mouth for 20 days. After receiving lactic acid starter culture, the product is stored in the refrigerator at 4 ° C for no more than 7 days before use. To preserve the obtained strains, 3 tubes of 1 mL of the obtained pure starter culture in SuproPlus 2640 were taken and stored at −75 ° C for up to a year.

### 2.5. Study Design

**Figure 1:**
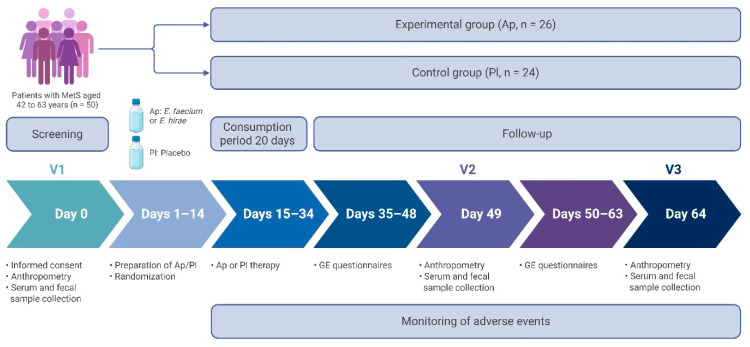
Study design. V1, V2 and V3 are visit designations corresponding to time points. Notes: Ap: Autoprobiotic; E. faecium: Enterococcus faecium; E. hirae: Enterococcus hirae; GE questionnaires: Gastroenterological questionnaires; MetS: Metabolic syndrome; Pl: SuproPlus 2640. Created with BioRender.com.

The study design is displayed in Figure 1. According to the protocol, the patients made three visits to the research center. At the first visit (V1), patients were assessed for compliance with the study’s inclusion and exclusion criteria, completed a survey of complaints, provided anamnesis, and underwent primary anthropometric measurements. The study participants also underwent an initial collection of biosamples, including serum for biochemical parameter measurements and feces for quantitative polymerase chain reaction (qPCR) and 16S rRNA gene sequencing. Subsequently, an autoprobiotic was prepared over 2 weeks (Days 1–14). After preparing the autoprobiotic starter culture, the patients were randomly divided into two groups: experimental (Ap) and control (Pl), who received either the autoprobiotic or SuproPlus 2640, respectively. The therapy duration was 20 days (Days 15–34), during which patients were unaware of whether they were receiving SuproPlus 2640 or an autoprobiotic. At the second visit (V2), which occurred 15 days after completing the course of autoprobiotic or SuproPlus 2640 therapy (Day 49), patients returned to the research center to remeasure anthropometric parameters and collect biosamples to assess laboratory parameters and fecal microbiota. The final visit (V3) was conducted 30 days after the end of autoprobiotic or SuproPlus 2640 intake (Day 64). During this visit, patients underwent a control measurement of anthropometric parameters and laboratory tests, as well as a fecal microbiota analysis. All materials collected at three visits were frozen and biobanked. Fecal samples were stored at −80 °C, and serum at −20 °C.

In the following description of the results, the study’s time points are labeled as Ap V1 or Pl V1 (Day 0), Ap V2 or Pl V2 (Day 49), and Ap V3 or Pl V3 (Day 64), respectively, based on the visit number and the group.

All patients were under the supervision of a therapist while taking the autobiotic and SuproPlus 2640. The doctors (gastroenterologist, endocrinologist, cardiologist) looked through daily questionnaires (for the presence of flatulence, pain, diarrhea, constipation, pain, arterial pressure, heart rate). Patients had the opportunity to receive on-line and off-line consultations with a doctor when their condition changed and complaints appeared. Participants were advised to maintain their usual dietary habits and physical activity levels in accordance with WHO guidelines on physical activity (at least 150–300 min of moderate-intensity aerobic activity per week) and AHA/NHLBI recommendations for metabolic syndrome management. Although participants received general lifestyle counseling based on WHO and AHA guidelines (www.heart.org, https://www.who.int/publications/i/item/9789240015128; https://www.ahajournals.org/doi/10.1161/circulationaha.105.169404, accessed on 10 July 2026) the lack of systematic monitoring of dietary intake and physical activity represents a limitation of this study.

### 2.6. Anthropometric Analysis

All patients included in the study had their body weight (BW) and BMI measured. Medical scales Massa-VEM-150 (Massa-K, St. Petersburg, Russia) were used to measure BW. BMI was calculated according to the methodology developed by Adolphe Quetelet, which is an indicator of the ratio of BW to height and is calculated individually according to the formula:

BMI (kg/m^2^) = BW (kg)/height (m^2^)

Additionally, the waist and hip circumferences, as well as the waist-to-hip ratio (WHR), were measured.

### 2.7. Gastroenterological Questionnaires

To assess the effect of autoprobiotic therapy on the gastrointestinal tract and the safety of their use (control of side effects), we used a self-developed questionnaire, which included the following symptoms: abdominal pain (0–4 points, where 0 is the absence of symptoms, 4 is the maximum manifestation), stool frequency (number of points = number of defecations per day), and flatulence and nausea (0–4 points, where 0 is the absence of symptoms, 4 is the maximum manifestation).

The Bristol stool scale was used to assess fecal shape and consistency. This scale describes seven types of feces: type 2 is characteristic of constipation, types 3 and 4 are considered “ideal stools”, and types 5, 6, and 7 are characterized by diarrhea.

### 2.8. Serum Biochemical Study

The biochemical study of serum samples was performed automatically using an Abbot ARCHITECT ci8200 (Holliston, MA, USA). The glucose and glycated hemoglobin levels were measured on an empty stomach. At the same time, the following lipid profile parameters were studied: total cholesterol, triglycerides (TG), high-density lipoproteins (HDL), low-density lipoproteins (LDL), very low-density lipoproteins (VLDL), non-HDL cholesterol, and the atherogenicity coefficient. All results were analyzed using the standards established in the clinical laboratory.

### 2.9. Study of the Gut Microbiota

Fecal samples were collected from patients to study their microbiota and delivered to the laboratory within 2 h after defecation. They were frozen immediately after delivery and stored at −80 °C.

#### 2.9.1. Quantitative Polymerase Chain Reaction (qPCR)

DNA was isolated from fecal samples (100 µL) using the Express-DNA-Bio reagent set for thermal lysis (Elkor Bio Company, St. Petersburg, Russian Federation). qPCR was performed using the Colonoflor 16 Premium kit (AlphaLab, St. Petersburg, Russian Federation) on a Mini-Opticon (BioRad, Hercules, CA, USA). All protocols were performed according to the manufacturer’s instructions.

This diagnostic kit allows for quantitative assessment of total bacterial count, bacteria from the *Enterobacteriaceae* family (*Escherichia coli*, bacteria belonging to the genera *Klebsiella*, *Salmonella*, *Proteus*, *Shigella*, *Citrobacter*, *Enterobacter*), *Bacteroides fragilis* group (BFG), other anaerobic bacteria, *Bacteroides thetaiotaomicron*, *Faecalibacterium prausnitzii*, *Prevotella* spp., *Roseburia inulinivorans*, *Eubacterium rectale*, *Clostridium perfringens*, *Clostridioides difficile*, *Akkermansia muciniphila*, *Fusobacterium nucleatum, Ruminococcus* spp., *Lactobacillaceae* family spp., *Bifidobacterium* spp*., Blautia* spp., *Methanobrevibacter smithii* (archaea), and fungi *(Candida* spp.).

#### 2.9.2. Bacterial DNA Extraction and Sequencing

Fecal samples (100 mg) were used for DNA extraction with the Hipure Soil DNA Kit (Magen Biotech, Guangzhou, China), according to the manufacturer’s instructions.

Raw paired-end 16S rRNA gene sequencing reads were processed in QIIME 2, version 2024.2. Primer sequences were removed using q2-cutadapt, and amplicon sequence variants were inferred using q2-dada2. Taxonomic classification was performed with a region-specific naïve Bayes classifier trained on SILVA release 138 reference sequences trimmed in silico to the V3–V4 region. Features classified as eukaryotic, mitochondrial, chloroplast, or unassigned at the domain level were excluded. Amplicon sequence variants were additionally taxonomically assigned against and NCBI 16S rRNA reference databases, and resulting names were reconciled with validly published taxa according to the List of Prokaryotic names with Standing in Nomenclature (LPSN). 

The 16S rRNA gene-based analysis was conducted following a previously described methodology [40]. Libraries representing the hypervariable V3 and V4 regions of the 16S rRNA gene were sequenced using the MiSeq platform (Illumina, San Diego, CA, USA). Genomic DNA was extracted from fecal samples employing the Hipure Soil DNA Kit (Magen Biotech). Library preparation followed the standard Illumina protocol, comprising two rounds of PCR. The first PCR step amplified a fragment of the 16S rRNA gene and appended adapter nucleotide sequences to the primers. The quality of the raw sequencing reads was assessed using FastQC.

Operational taxonomic units (OTUs) were identified using CD-HIT-OTU-MiSeq with the following parameters: the lengths of high-quality segments of R1 and R2 reads were set at 200 and 180 base pairs, respectively; a 97% similarity threshold was used for clustering; and an abundance cutoff of 0.00001 was applied. Taxonomic annotation of OTUs was performed using the Greengenes database (version 13.5). The CD-HIT-OTU-MiSeq pipeline enables OTU identification from terminal read segments without requiring paired-end read merging.

Raw paired-end 16S rRNA gene sequencing reads were processed in QIIME 2, version 2024.2. Primer sequences were removed using q2-cutadapt, and amplicon sequence variants were inferred using q2-dada2. Taxonomic classification was performed with a region-specific naïve Bayes classifier trained on SILVA release 138 reference sequences trimmed in silico to the V3–V4 region. Features classified as eukaryotic, mitochondrial, chloroplast, or unassigned at the domain level were excluded.

Microbiome analysis was performed using the ASV/feature abundance table exported in BIOM format. Taxonomic features were converted to numeric values, negative values were set to zero where applicable, and relative abundance was calculated by dividing each feature by the total feature sum per sample. Alpha diversity was assessed using observed ASVs/features, Shannon diversity, Simpson diversity, inverse Simpson index, evenness, and Chao1 where available. Beta diversity was assessed using Bray–Curtis, Jaccard, and Aitchison distances. Principal coordinate analysis was used for visualization. 

### 2.10. Statistical Analysis

As this study was designed as a pilot and exploratory investigation to evaluate the feasibility and preliminary effects of a novel personalized autoprobiotic intervention, no formal a priori sample size calculation or statistical power analysis was performed. The sample size was determined based on the strict inclusion/exclusion criteria, the availability of eligible participants, and the logistical constraints associated with the production of individualized autologous strains. Consequently, the findings of this study should be considered hypothesis-generating.

To assess the normality of the data distribution, the Kolmogorov–Smirnov test was conducted, which indicated the suitability of nonparametric statistical methods. Statistically significant differences between groups were determined using the Mann–Whitney U test, with the Benjamini–Hochberg procedure applied to adjust for multiple comparisons. Additionally, the Wilcoxon signed-rank test was employed for analyses involving paired samples.

For comparative analysis, a post hoc test of honest significant difference for unequal N was used in Statistica 10.0. Differences with *p* < 0.05 were deemed significant. Exploring correlations among the studied parameters, we used Spearman’s rank correlation test in Statistica 10.0 (StatSoft, Tulsa, OK, USA).

Vectors for principal component analysis using PERMANOVA were based on OTU abundances, filtered for noise, and normalized for total OTU counts in each sample. PERMANOVA was used to assess the association of study group and visit with beta diversity structure. *p*-values were adjusted for multiple testing using the Benjamini–Hochberg method, where applicable.

Data processing and analysis were performed using R version 4.4.0. The Shannon, Simpson, and Chao1 biodiversity indices were calculated using the vegan package (v. 2.6-10) [55]. PERMANOVA was used to assess the association of study group and visit with beta diversity structure. P-values were adjusted for multiple testing using the Benjamini–Hochberg method where applicable.

The ‘coda4microbiome’ version 0.2.4 was used for longitudinal analysis of microbiota changes over time [56], designed to analyze microbiome data that account for compositional properties and longitudinal structure. The taxa were aggregated to the genus level using regular expressions and then normalized to obtain the overall microbiota composition. Recurring taxa were merged. Correlation analysis of quantitative indicators at time point V1 was performed using Spearman’s rank correlation. Significant results (*p* < 0.05) were visualized using the ggplot2 package.

The linear mixed-effects models were used to analyze longitudinal outcomes measured at visits V1, V2, and V3, incorporating fixed effects for treatment group, visit, and their interaction, as well as a random intercept for participant. The group × visit interaction was considered the primary statistical test for differences in trajectories between the autoprobiotic and control groups. For each outcome, estimated marginal means, between-group differences at each visit, within-group changes from V1, and difference-in-differences contrasts for V2−V1 and V3−V1 were additionally calculated. All effects are reported as effect estimates with 95% confidence intervals and *p*-values. Responder/non-responder analyses and the exploration of factors associated with differences in individual response were considered exploratory and hypothesis-generating. 

### 2.11. Study Limitations

The limitations of the study were as follows: (1) a small number of patients in each group (small sample size increased risk of errors and needed for cautious interpretation of the results); (2) insufficient control over diet and physical activity; (3) the nutrient medium SuproPlus 2640 used in the control group Pl is not biologically inert; (4) insufficient characterization of the genomic safety of autoprobiotic strains; (5) lack of evidence of persistence of autoprobiotic strains; and (6) this study did not include whole-genome sequencing of the *Enterococcus* strains used, and was not designed for direct strain-tracking after administration of the autoprobiotic.

## 3. Results

### 3.1. Clinical Data

To visualize the progress of participants on the scale of a parallel randomized study, we summarized them in terms of stages, including registration; grouping; observation; and analysis of the information received. The purpose of this approach was to demonstrate the number of participants at each individual stage, from the moment of registration to summing up the results, as well as the quantity of sampled biological material (blood serum and feces) (Figure S1,Tables S1 and S2, section Supplementary Materials).

#### 3.1.1. Anthropometry

A decrease in anthropometric parameters was observed in the Ap group after 20 days of autoprobiotic use (Table 2). These patients had decreased BW, BMI, waist circumference, and hip circumference. We also saw a decrease in WHR in the Ap group.

In the control group, only a short-term reduction in waist circumference at the time point Pl V2 was detected, and no other significant changes in anthropometric parameters were found. It should be noted that the changes observed in patients from the Ap group were not short-term, as they persisted 30 days after the end of autoprobiotic therapy.

It should be noted that these changes were not found in all patients treated with an autoprobiotic. No changes in BW and BMI were found in 4 patients from the Ap group. Waist circumference and hip circumference did not decrease in 3 and 6 patients out of 26, respectively.

The maximum range of therapy effects was observed in the Ap group; particularly for patient BW, which ranged from 1 to 11 kg (at the ApV1 and ApV3 time points). Comparing the medians of the anthropometric study indicators revealed decreases of 2 kg in BW, 0.8 kg/m^2^ in BMI, and 4 cm and 2 cm in waist and hip volume, respectively.

#### 3.1.2. Gastroenterological Questionnaires and Safety

In the analysis of gastroenterological questionnaires, no serious adverse events (AEs) were identified among study participants. Patients did not have symptoms such as abdominal pain, nausea, or abdominal bloating. Additionally, most patients reported decreased flatulence and improved stool quality. The trial recorded 12 AEs, classified as mild or moderate. The number of patients with AEs was similar with autoprobiotics and SuproPlus 2640: 7 AEs were recorded in the Ap group and 5 in the control group. Two mild AEs (heartburn and belching) were classified as possibly related to the study product in the Ap group and resolved within 2 and 1 days, respectively; one mild AE (belching) was classified as possibly related to SuproPlus 2640 and was resolved by the end of the study. The remaining AEs were deemed unrelated to the interventions. There were no discontinuations due to AEs in either group. Thus, Ap was considered safe and well-tolerated.

### 3.2. Biochemical Parameters

#### 3.2.1. Carbohydrate Metabolism

**Figure 2:**
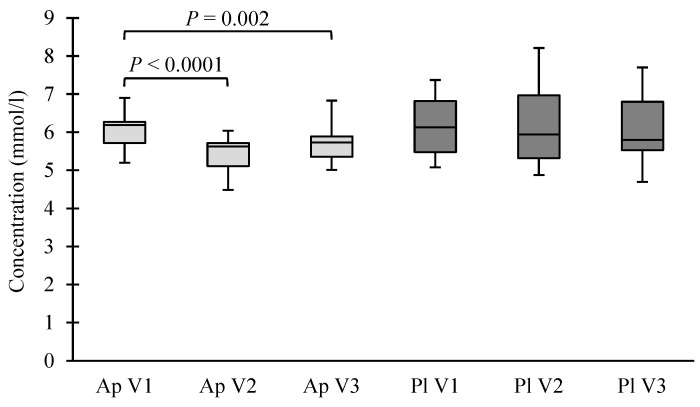
The serum blood glucose concentration of patients in the Ap and Pl groups at different time points. Notes: The experimental (Ap) group and the control (Pl) group consisted of patients with metabolic syndrome who received an autoprobiotic or SuproPlus 2640, respectively. Time points are labeled as Ap V1 or Pl V1 (Day 0), Ap V2 or Pl V2 (Day 49), and Ap V3 or Pl V3 (Day 64). Days 15–34 represent the period of autoprobiotic or SuproPlus 2640 consumption. Results are presented as median (25%; 75%).

Serum glucose levels were generally at the upper limit of the reference range; however, after the introduction of the autoprobiotic, glucose concentrations decreased significantly in the Ap group, whereas they remained unchanged in the Pl group (Figure 2).

**Figure 3:**
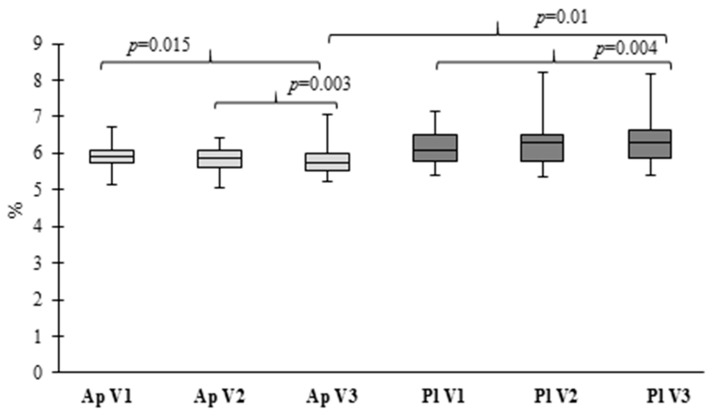
The concentration of glycated hemoglobin in the blood serum of patients from the Ap and Pl groups at different times of the experiment. Notes: experimental (Ap) and control (Pl) groups, patients with MetS, who received an autoprobiotic or SuproPlus 2640, respectively. Control check points Ap V1 or Pl V1 (0 day), Ap V2 or Pl V2 (49th day), Ap V3 or Pl V3 (64th day). 15–34 days—autoprobiotic or SuproPlus 2640 therapy. Results are presented as median (25%; 75%).

Glucose levels decreased after autoprobiotic consumption in almost all (24 out of 26) patients in the Ap group, except for two patients. At the same time, the median changes amounted to 0.54 mmol/L when comparing the time points Ap V1 and Ap V3. The minimum and maximum changes were 0.4 mmol/L and 5.0 mmol/L, respectively. The content of glycated hemoglobin in most cases was at the upper limit of the norm; however, after the introduction of the autoprobiotic, the concentrations of these substances significantly decreased in the Ap group, in contrast to the Pl group (Figure 3). It was noteworthy that an intergroup comparison revealed lower concentrations of HbAc1 and triglycerides in the blood serum when comparing samples taken from Ap and Pl groups on day 28 after therapy.

#### 3.2.2. Lipid Profile

After using the PFFP, positive changes in the lipid profile were observed in patients with MetS (group Ap). The levels of the main atherogenic fractions—namely, total cholesterol, TG, and VLDL cholesterol—decreased (Table 3). It is also worth noting that increases in proatherogenic lipids and HDL cholesterol were observed. Among patients receiving SuproPlus 2640, no significant changes in the lipid profile were observed.

Effects on lipid metabolism were observed in most (22 out of 26) patients in the Ap group, with variations across individual indicators. Total cholesterol decreased to a median of 0.5 mmol/L (19.3 mg/dL) in 20 out of 26 patients, with changes ranging from 0.3 to 3.4 mmol/L (11.6–31.4 mg/dL).

VLDL levels did not decrease in 8 patients, ranging from 0.1 to 1.2 mmol/L (3.9–46.4 mg/dL), with a median difference of up to 0.41 mmol/L (15.85 mg/dL). The median changes in TG and HDL were 0.5 mmol/L (43.75 mg/dL) and 0.2 mmol/L (17.5 mg/dL), respectively. It should be noted that an intergroup comparison revealed lower concentrations of triglycerides in the blood serum when comparing samples taken from Ap and Pl groups on day 28 after therapy. This parameter decreased when comparing ApV1 and ApV3 in 15 patients out of 26 in the Ap group, and only one out of 24 patients in the control group.

### 3.3. Gut Microbiota Study

#### 3.3.1. Quantitative Polymerase Chain Reaction

**Figure 4:**
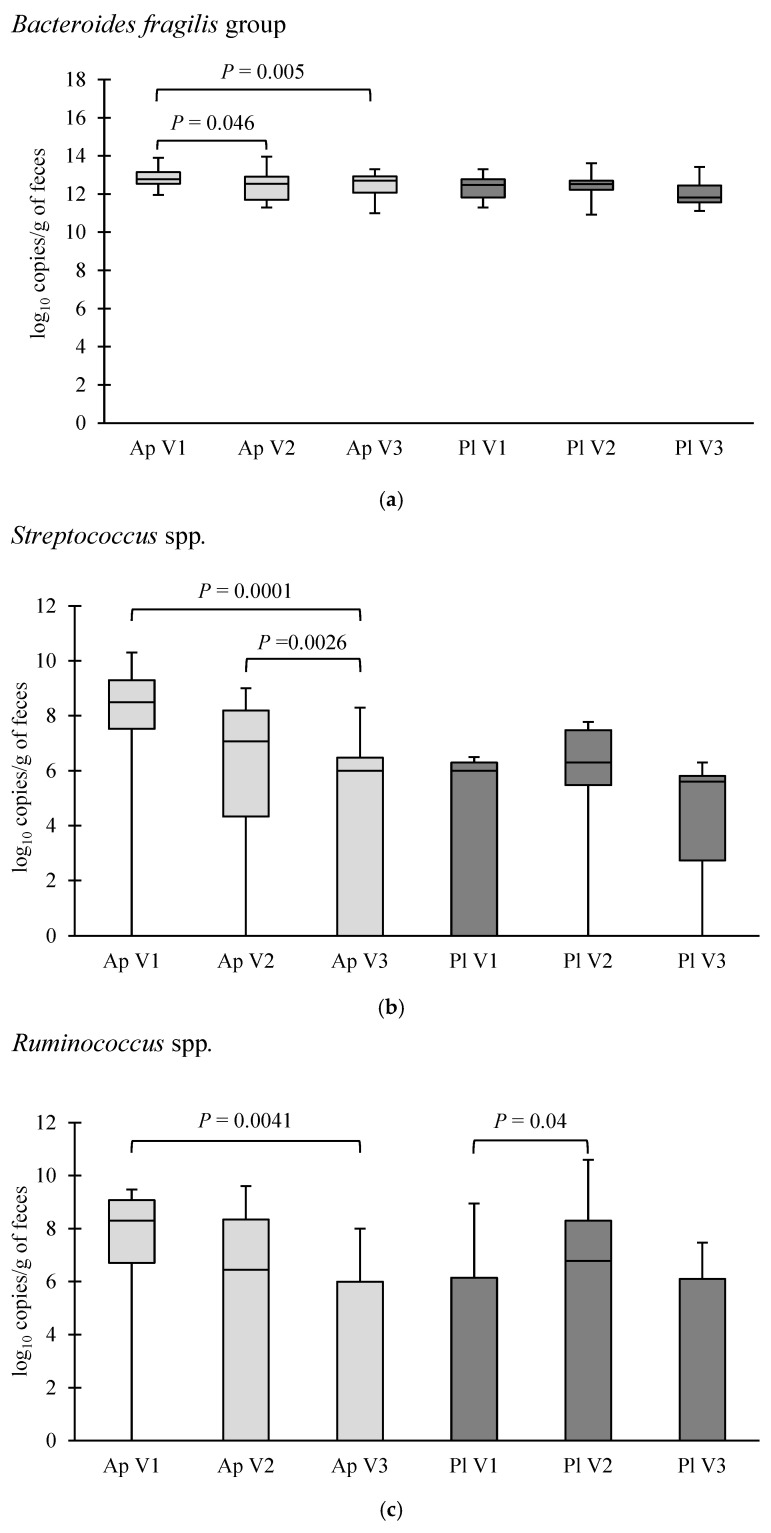
The numbers of *Bacteroides fragilis* group (**a**), *Streptococcus* spp. (**b**), and *Ruminococcus* spp. (**c**) in the fecal microbiota of patients in the Ap and Pl groups at different time points. Notes: The experimental (Ap) group and the control (Pl) group consisted of patients with metabolic syndrome, who received an autoprobiotic or SuproPlus 2640, respectively. Time points are labeled as Ap V1 or Pl V1 (Day 0), Ap V2 or Pl V2 (Day 49), and Ap V3 or Pl V3 (Day 64). Days 15–34 represent the period of autoprobiotic or SuproPlus 2640 consumption. Results are presented as median (25%; 75%).

Only in the Ap group was a consistent (when comparing time points Ap V1, Ap V2, and Ap V3) decrease in BFG (Figure 4a) observed. Similar changes were observed in the numbers of *Streptococcus* spp. and *Ruminococcus* spp., with reductions in their quantitative contents only in the Ap group, and no change in the Pl group (Figure 4b,c).

Oppositely directed changes were found in the analysis of the *Ruminococcus* spp. population (Figure 4c). In the Ap group, a reduction in the number of *Ruminococcus* spp. was observed when comparing Ap V1 and Ap V3 time points. In contrast, the Pl group showed an increase in *Ruminococcus* spp. numbers by the second visit (the time point Pl V2).

#### 3.3.2. 16S rRNA Gene Sequencing

The ASV/feature (Table S1 in Supplementary Materials section) was obtained from the BIOM file. The table was used for diversity analysis, ordination, and exploratory differential abundance screening. Features with low prevalence were filtered where required for visualization and exploratory modeling. The analysis of the microbiome for alpha diversity and beta diversity did not reveal significant changes in the composition of the microbiomes of all patients, regardless of the group and visits, but only reflected the personal characteristics of the patients.

The 16S rRNA microbiome study did not reveal significant alpha diversity in fecal samples of all patients, regardless of the group and visit, but only reflected the personal characteristics of the patients (Figure S2 in section of Supplement Materials).

The longitudinal model revealed statistical evidence of differences in trajectories between groups for individual alpha diversity indicators, primarily Chao1 and observed taxa (Tables S6–S10, Supplementary Materials). However, practical interpretation should be based on the magnitude of the effect and the 95% CI, rather than solely on the *p*-value. For observed taxa, the V3−V1 contrast between Ap and Pl indicated a more favorable trend in the Ap group (difference-in-differences estimate ≈ 5.31; 95% CI ≈ 0.24–10.37). For Chao1, the group × visit omnibus test is significant, but the individual difference-in-differences contrasts have wider confidence intervals; therefore, this result should be considered as a statistical signal that requires confirmation in a larger study.

Individual variability in the microbiome response was further examined in an exploratory responder/non-responder analysis. An increase in the Shannon index from V1 to V3 was used as a preliminary criterion for response. According to this definition, response was observed in 5/12 Ap participants (41.7%) and in 0/6 Pl participants (0.0%); the intergroup difference did not reach statistical significance according to the Fisher exact test (*p* ≈ 0.114). This analysis was considered hypothesis-generating and was not used as primary evidence of the effectiveness of the intervention (Tables S5 and S6 in section of Supplement Materials).

Beta-diversity analysis (Figure S3A–D in the Supplementary Materials) demonstrated a statistically significant association between the combined group/visit model and microbiome composition. PERMANOVA showed that the model explained 13.4% of variation in Bray–Curtis distances (R^2^ = 0.134, *p* = 0.001), 10.8% of variation in Jaccard distances (R^2^ = 0.108, *p* = 0.020), and 10.8% of variation in Aitchison distances (R^2^ = 0.108, *p* = 0.024). These findings indicate that the microbiome composition differed across the analyzed study groups and/or visits. Bray–Curtis, Jaccard, and Aitchison distances were used to assess the structure of the microbiota. Baseline-only PERMANOVA did not reveal a significant initial separation between the Ap and Pl groups at V1. In longitudinal PERMANOVA, the term group × visit was of primary interest, but it did not provide consistent statistical evidence of differences in trajectories between groups based on the presented beta diversity metrics (Tables S11–S14, Supplementary Materials). The distance-from-baseline analysis also did not reveal significant intergroup differences. Therefore, the beta diversity results describe the overall variability of the microbiota and the visual characteristics of individual trajectories, rather than providing standalone proof of intervention effectiveness.

**Figure 5:**
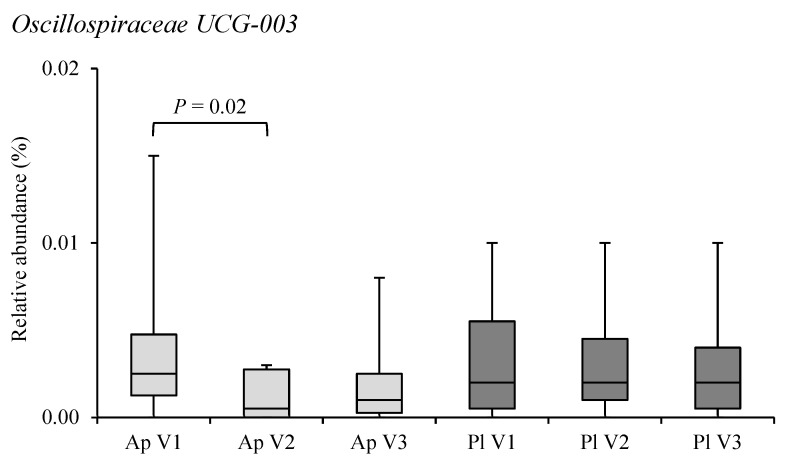
The relative abundance of genus *Oscillospiraceae UCG-003* in the fecal microbiota of patients in the Ap and Pl groups at different time points. Notes: The experimental (Ap) group and the control (Pl) group consisted of patients with metabolic syndrome, who received an autoprobiotic or SuproPlus 2640, respectively. Time points are labeled as Ap V1 or Pl V1 (Day 0), Ap V2 or Pl V2 (Day 49), and Ap V3 or Pl V3 (Day 64). Days 15–34 represent the period of autoprobiotic or SuproPlus 2640 consumption. Results are presented as median (25%; 75%).

**Figure 6:**
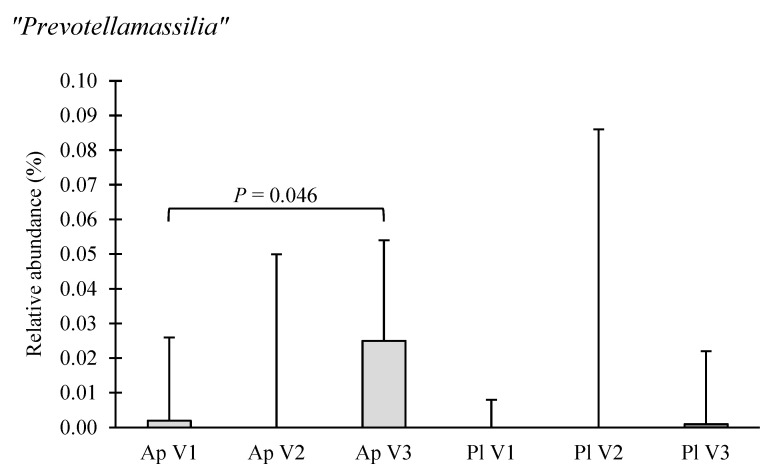
The relative abundance of “*Prevotellamassilia*” in the fecal microbiota of patients in the Ap and Pl groups at different time points. Notes: The experimental (Ap) group and the control (Pl) group consisted of patients with metabolic syndrome who received an autoprobiotic or SuproPlus 2640, respectively. Time points are labeled as Ap V1 or Pl V1 (Day 0), Ap V2 or Pl V2 (Day 49), and Ap V3 or Pl V3 (Day 64). Days 15–34 represent the period of autoprobiotic or SuproPlus 2640 consumption. Results are presented as median (25%; 75%).

The 16S rRNA gene sequencing revealed a decrease in the relative abundance of the genus *UCG-003*, belonging to the family *Oscillospiraceae* (*Ruminococcaceae*) (Figure 5), in fecal samples collected on the 14th day after the end of the autoprobiotic treatment (time point Ap V2). There were no significant changes in this parameter at the Ap V3 time point. At the same time, the relative abundance of the genus “*Prevotellamassilia*”, belonging to the family *Prevotellaceae,* increased when comparing time points Ap V1 and Ap V3 (Figure 6).

### 3.4. Compositional Analysis of the Microbiome

**Figure 7:**
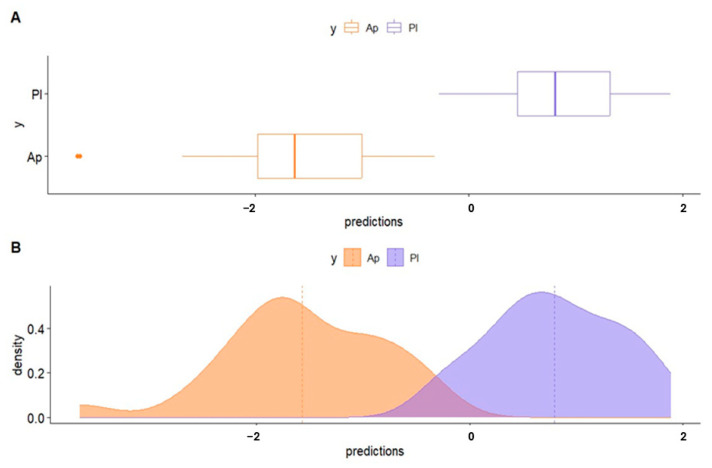
Forecast graph: a comparative analysis of predicted value distributions (**A**) and density curves of predicted value distributions (**B**) for the Ap and Pl groups. Notes: The experimental (Ap) and control (Pl) groups include patients with metabolic syndrome who received either an autoprobiotic or SuproPlus 2640, respectively. Graph A displays boxplots of predicted value distributions for both groups, showing medians and the 25–75% interquartile range. Graph B illustrates the density curves for predicted value distributions, with the orange curve representing the Ap group and the violet curve representing the Pl group.

The total changes in the microbiome were analyzed using a compositional analysis, comparing the Ap and Pl groups (Figure 7).

Graph A shows the prediction values for the Ap and Pl groups. The Pl group has predicted values, on average, close to zero or higher (up to 1.9), which may indicate low variability and greater uniformity in the SuproPlus 2640 group. By contrast, the Ap group in this graph showed significantly negative values (as low as −3.8), indicating a substantial difference relative to SuproPlus 2640.

### 3.5. Most Significant Taxa

**Figure 8:**
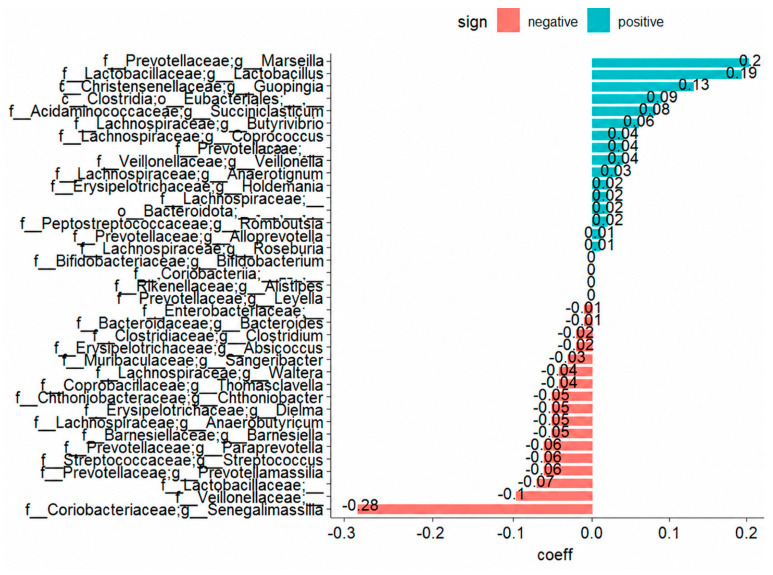
Signature Graph: Taxonomic feature importance by treatment group over time. Notes: The graph illustrates the significance of individual taxa within a predictive model comparing the two groups of patients with metabolic syndrome, those receiving autoprobiotics (Ap) and those receiving SuproPlus 2640 (Pl). Positive coefficients (blue bars) indicate taxa associated with the Pl group, while negative coefficients (red bars) indicate taxa related to the Ap group.

The taxa that had a significant impact on the model when comparing the two groups were also analyzed (Figure 8).

Positive coefficients in the blue columns indicate taxa more closely associated with SuproPlus 2640 therapy, meaning they have a higher relative abundance in patients receiving SuproPlus 2640 therapy. In turn, negative coefficients in the red columns indicate taxa related to the autoprobiotic treatment, meaning they have increased relative abundance in the Ap group.

The most significant taxa associated with SuproPlus 2640 were the genus “*Marseilla*” (*Prevotellaceae*) (coefficient 0.2), *Lactobacillus* (coefficient 0.19), and *Guopingia* (*Christensenellaceae*) (coefficient 0.13). These taxa were not directly exposed to autoprobiotic therapy or to components of SuproPlus taken by patients in the Pl group. At the same time, the most significant taxa associated with autoprobiotics include the genus *Senegalimassilia* (*Eggerthellaceae*) (coefficient −0.28), the families *Veillonellaceae* (coefficient −0.1) and *Lactobacillaceae* (coefficient −0.07), and the genera “*Prevotellamassilia*”, *Streptococcus*, *Paraprevotella*, and *Anaerobutyricum* (coefficient −0.06), indicating their growth in response to Ap therapy.

### 3.6. Correlation Analysis

#### 3.6.1. Correlation Analysis Between Bacterial Taxa

**Figure 9:**
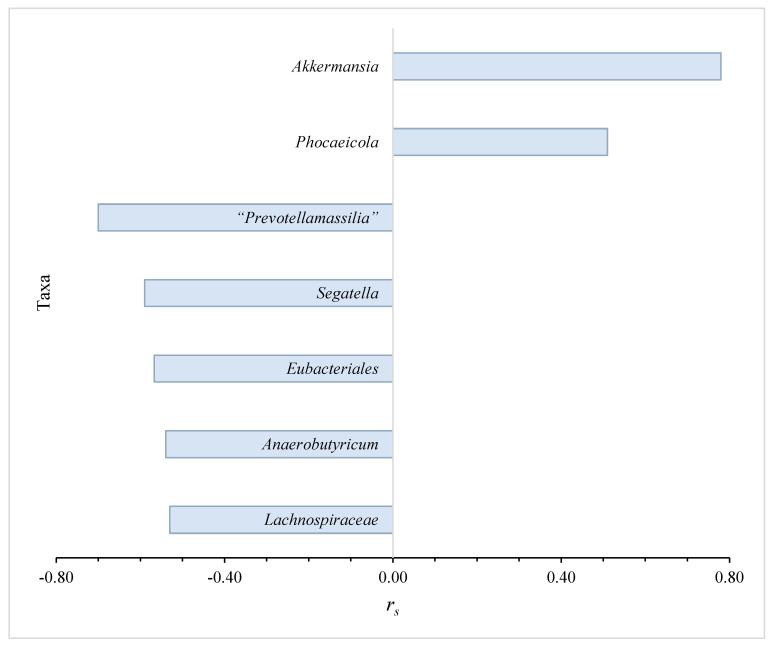
Correlation coefficients between the numbers of *Bacteroides fragilis* group and the relative abundance of specific taxa in the fecal microbiota of patients in the Ap group. Notes: r_s_: Spearman’s rank correlation coefficient. *p* < 0.05 for all correlations.

Correlation analysis of the results of the microbiome study (Figure 9, Figure S4 in Supplementary Materials section) revealed negative correlations only between the numbers of BFG and the relative abundance of the genera *Anaerobutyricum*, “*Prevotellamassilia*”, and *Segatella*. At the same time, a positive correlation was shown between the numbers of BFG and the relative abundance of the genus *Phocaeicola*.

#### 3.6.2. Correlation Analysis Between Bacterial Taxa Abundance and Laboratory Parameters

**Figure 10:**
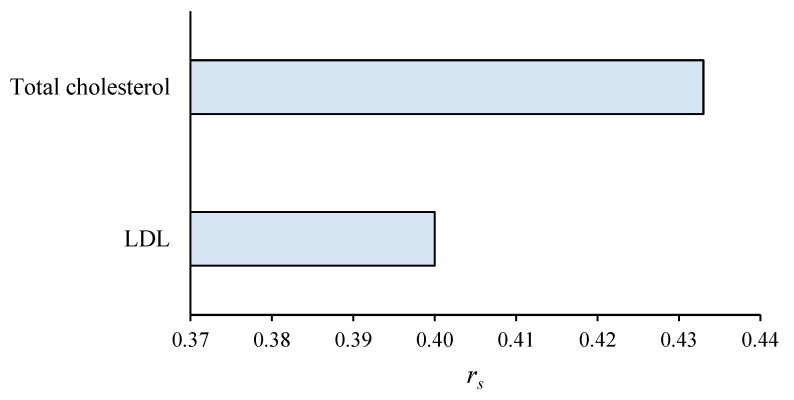
Correlation coefficients between the numbers of *Bacteroides fragilis* group and parameters of the serum lipid profile of patients in the Ap group. Notes: Ap: Autoprobiotic; LDL: Low-density lipoproteins; r_s_: Spearman’s rank correlation coefficient. *p* < 0.05 for all correlations.

A positive correlation was observed between the number of BFG and the concentrations of LDL and total cholesterol in the serum of patients in the Ap group (Figure 10).

## 4. Discussion

In the present study, the effectiveness of autoprobiotics—non-pathogenic indigenous bacteria isolated from patients’ gut and subsequently introduced to the same patients as a PFFP—was evaluated. In this pilot clinical trial, the effects of autoprobiotic *Enterococcus faecium* and *Enterococcus hirae* strains in the treatment of MetS, characterized by obesity and impaired carbohydrate and lipid metabolism, were investigated.

The control group of patients received SuproPlus2640, which served as a nutrient medium for cultivating autoprobiotic enterococcal strains. A critical methodological consideration in the present study is the nature of the control intervention. While initially conceptualized as a placebo-controlled trial, the control group received the cell-free SuproPlus 2640 culture medium used as the vehicle for the autoprobiotics. SuproPlus 2640 is not an inert placebo, and its use proved to be successful. In this way, an assumption about the importance of treatment with an autoprobiotic grown on a nutrient medium that contains prebiotic components could be avoided.

However, it is important to emphasize that both the autoprobiotic and the control groups received the exact same volume and composition of the culture medium. Because the vehicle was strictly controlled across both arms, the statistically significant differences observed in the gut microbiota composition and metabolic parameters between the two groups are highly likely attributable to the biological activity of the live autologous *Enterococcus* strains, rather than the vehicle itself. Nevertheless, the potential biological activity of the culture medium and the inability to include a completely inert control (e.g., sterile saline) remain significant methodological limitations of this study. These factors must be taken into account when interpreting the absolute magnitude of the observed effects, and future study designs should explore the use of inert suspension vehicles (if clinically and microbiologically feasible).

Autoprobiotics were administered for a short duration (20 days), which contrasts significantly with the longer regimens (8–12 weeks) typically recommended for treating MetS with probiotics [57,58]. The assumption regarding the long-term preservation of the autoprobiotic effect was justified in our study. All the effects of the autoprobiotic were noted 14 and 28 days after discontinuation of therapy. This sustained effect may be attributed to the prolonged presence of a substantial population of the introduced indigenous bacteria. The results of this clinical trial were consistent with our previous findings in experimental dysbiosis in Wistar rats. In those experiments, recombinant erythromycin-resistant indigenous *Enterococcus* strains were administered to rats for three days. These strains persisted in the intestines for more than 24 days, as confirmed by bacteriological analysis of fecal samples [59].

As noted in the introduction, the use of various probiotic strains or their mixtures in MetS therapy has shown inconsistent clinical efficacy, with some studies reporting normalization of specific anthropometric and biochemical serum parameters, or statistically significant reductions in BW, glucose levels, and lipid profile components [42]. In most of the same studies, an analysis of gut microbiome changes was not included. A previously published meta-analysis of probiotic food and supplement interventions, which included 18 randomized controlled trials that met at least one criterion for MetS, highlighted these disparities [58].

In our study, a 20-day course of autoprobiotic *Enterococcus* strains in patients with MetS led to reductions in BW, BMI, and waist and hip circumferences in the autoprobiotic group (Ap), compared to the SuproPlus 2640 group (Pl). Specifically, the median BMI decreased by 0.8 kg/m^2^ (with a median BW loss of 2.3 kg). Such substantial changes have not been observed in previous probiotic studies, in which reported reductions in BMI ranged from 0.27 kg/m^2^ to 0.6 kg/m^2^ and were not clinically significant [60].

It is worth noting the significant decrease in WHR in the Ap group. The WHR is a more reliable indicator of metabolic health and cardiometabolic risk than BMI [61]. This result is particularly noteworthy because a recent systematic review of meta-analyses found that, in prior studies, the effects of probiotic therapy on WHR were not statistically significant [62].

We also observed a reduction in fasting serum glucose concentrations, with a median decrease of 0.54 mmol/L. This is comparable to a meta-analysis of probiotic therapy in T2DM, where glucose levels were reduced by 0.5 mmol/L [58]. Changes in lipid profiles were observed in most patients, with total cholesterol levels decreasing by 0.3–3.4 mmol/L (11.6–131.4 mg/dL) and a median reduction of 0.5 mmol/L (19.3 mg/dL).

Other studies have reported partial improvements in lipid profiles, though these changes were inconsistent across probiotic types and were generally limited to statistically significant but clinically marginal effects. In a meta-analysis of probiotics in patients with MetS, the total cholesterol concentration decreased by only 0.16 mg/dL (0.004 mmol/L) [58]. It was especially important to detect a difference between the Ap and Pl groups in terms of triglycerides at one month after therapy.

In our study, we demonstrated a statistically significant decrease in glycated hemoglobin (HbA1c) levels just one month after the initiation of autoprobiotic therapy in patients with MetS, when compared to the control group. This finding warrants specific discussion, as the standard clinical guideline recommends evaluating HbA1c no earlier than three months post-intervention, given the average lifespan of erythrocytes (approximately 120 days). Nevertheless, the early reduction in HbA1c observed in our study is physiologically sound. According to the kinetics of non-enzymatic glycation, the most recent 30 days exert a dominant influence, contributing approximately 50% to the final HbA1c value. Therefore, assuming a substantial and rapid decline in plasma glucose concentrations during the first weeks of probiotic administration, this inevitably slows the glycation rate of circulating red blood cells and becomes detectable in laboratory parameters by the end of the first month. The rapid metabolic response observed in our work is likely linked to the specific effects of autoprobiotics on the pathophysiology of MetS.

All observed clinical and laboratory changes in this study coincided with alterations in the gut microbiota, which were assessed comprehensively using qPCR and 16S rRNA gene-based analysis.

We did not aim to characterize the specific microbiome features associated with MetS, as this remains a complex and unresolved issue despite the relatively large number of studies. The inconsistent results of microbiome analyses in MetS are likely due to variations in patient demographics, including geographic location, diet, age, sex, and MetS manifestations, as well as differences in sample collection, handling, and research methods.

The qPCR analysis in the Ap group revealed a significant decrease in the numbers of BFG members, *Streptococcus* spp., and *Ruminococcus* spp.

The 16S rRNA microbiome study did not reveal significant alpha and beta diversity in fecal samples of all patients, regardless of the group and visit, but only reflected the personal characteristics of the patients (Figures S1 and S2 in the Supplementary Materials). In fact, the beta diversity analysis and PERMANOVA showed that the microbiome composition differed across the analyzed study groups and/or visits. Indeed, the revealed variance (R2) at the level of 10–13% is considered a moderate and biologically significant effect. However, it is worth noting that most of the variations (>86%) remain unexplained by this model. This is typical for microbiome studies and indicates the influence of many other unaccounted-for covariates (for example, individual patient characteristics, statin and antihypertensive medications, and other causes of difficult-to-control medications or genetic background), which determines the need for future longitudinal studies with more detailed metadata collection.

The longitudinal model revealed statistical evidence of differences in trajectories between groups for individual alpha diversity indicators, primarily Chao1 and observed taxa (Tables S6–S8, Supplementary Materials). For the observed taxa, the V3−V1 contrast between Ap and Pl indicated a more favorable trend in the Ap group.

At the same time, an interesting finding in the study was the changes in relative abundance of bacteria belonging to the phylum *Bacteroidota.* Although this trait was once considered a marker of T2DM and obesity, its reliability is increasingly questioned due to the heterogeneity within this taxon. Increases in the population of *Bacteroidaceae*, including *Bacteroides* spp., have already been detected in qPCR and metagenomic studies [63].

Currently, the BFG includes two families (*Bacteroidaceae* and *Tannerellaceae*) with several genera. This group contains approximately 20 distinct species, with *Bacteroides*, *Parabacteroides*, and *Phocaeicola* species being the most common in anaerobic human infections. Clinically relevant species include *B. fragilis*, *B. thetaiotaomicron*, *Parabacteroides distasonis*, *Phocaeicola vulgatus* (basonym: *Bacteroides vulgatus*), *B. ovatus*, and *B. uniformis*. While BFG species are key residents of healthy gut microbiomes and confer various beneficial effects, they are also frequently isolated from anaerobic infections, reinforcing their role as true pathobionts [64].

The main changes in the Ap and Pl groups were associated with different combinations of taxa. To analyze microbiome data, we employed a fundamentally new bioinformatics method, coda4microbiome, which was developed to identify a model with a minimum number of features while maximizing predictive power [65]. Using coda4microbiome, we identified a microbial signature with maximum discrimination accuracy between the Pl and Ap groups. The signature was determined by the relative abundance of two groups of taxa (Figure 7). The Pl group was dominated by three taxa, the sum of the coefficients of which was 0.52: “*Marseilla*” (*Prevotellaceae*), *Lactobacillus* (*Lactobacillaceae*), and *Guopingia* (*Christensenellaceae*). In the Ap group, four taxa were dominant with a coefficient sum of −0.51: *Senegalimassilia* (*Eggerthellaceae*), *Veillonellaceae*, *Lactobacillaceae*, and “*Prevotellamassilia*” (*Prevotellaceae*).

The genus *Senegalimassilia* (coefficient −0.28), positively associated with Ap therapy, contributed the most to the separation of the Ap and Pl groups. Previously, it was shown that *Senegalimassilia* is negatively associated with MetS in patients with schizophrenia [66]. In this case, this taxon was identified as the only microbial marker of MetS. The relative abundance of *Senegalimassilia* decreased with MetS aggravation, as well as in children with excess BW; the taxon was inversely associated with inflammation markers, hepato-visceral fat, and type 2 diabetes [66]. The relative abundance of *Senegalimassilia* was reduced in patients with diabetic retinopathy [67] and adolescent girls with polycystic ovary syndrome [68], and is negatively associated with NAFLD [69]. Analysis of the causal relationship between the gut microbiome and hypertension in a bidirectional Mendelian randomization trial showed that *Senegalimassilia* protects against the development of hypertension and is statistically more protective than other taxa [70]. *Senegalimassilia* was negatively associated with the risk of endometriosis, an estrogen-dependent disease whose complex pathogenesis includes metabolic changes [71]. In addition, *Senegalimassilia* can produce enterolactone (an organic compound with anti-inflammatory properties derived from dietary lignins) [72] and metabolize dietary polyphenols [73].

Based on the literature and our results, we hypothesized that *Senegalimassilia*, along with BFG species, may be a potential biomarker of the response to microbiome-modulating therapies, such as autoprobiotics, in MetS and other cardiometabolic diseases (CMD).

*Veillonellaceae* is a heterogeneous family of commensal succinate-, acetate-, and propionate-producing bacteria [74]. The abundance of *Veillonellaceae* was reduced in patients with NAFLD [75]. Additionally, the family *Veillonellaceae* was inversely associated with hyperglycemia and serum levels of the proinflammatory cytokine IL-6 in patients with T2DM [76]. *Lactobacillaceae* is a well-studied family of metabolically active bacteria that, in most cases, have probiotic properties [77].

“*Prevotellamassilia*”, the relative abundance of which was also increased by Ap therapy and was inversely associated with the proinflammatory BFG, may represent a potential probiotic taxon from the *Prevotellaceae* family. A decrease in the relative abundance of “*Prevotellamassilia*” has previously been shown in T2DM [78]. In addition, in an experimental study in mice, “*Prevotellamassilia*” mediated the lipid-lowering effect of green tea [79]. In another study, the abundance of *“Prevotellamassilia”* and other beneficial bacteria was increased by supplementation with dietary bacterial cellulose, which improved metabolic health in mice on a high-fat diet [80].

The genus *Oscillospiraceae UCG-003* (synonym: *Ruminococcaceae UCG-003*), which was significantly reduced with Ap therapy compared to Pl, although did not appear in the differentiating signature, was previously identified as a marker taxon in humans prone to obesity (abundance increased) [81]. A negative association was also found between the change in HDL cholesterol and the change in *Ruminococcaceae UCG-003* after a 6-week combined exercise and diet intervention that reduced BW and improved central hemodynamics in adolescents with obesity [82]. In mice, *Ruminococcaceae UCG-003* was positively correlated with markers of intestinal barrier damage (NF-κB mRNA, TLR4 mRNA, TLR5 mRNA, endotoxin), and its decrease with polyphenol intake was associated with the prevention of NAFLD development [83]. The relative abundance of *Oscillospiraceae UCG-003* was increased in mice on a high-fat diet and then decreased with the intake of gac aryl polysaccharides, which have the potential to treat obesity and metabolic dysfunction [84]. However, other studies have shown that *Ruminococcaceae UCG-003* was significantly inversely associated with some CMD [85], which may indicate its potential beneficial effects. Thus, there is evidence for an association between *Oscillospiraceae UCG-003* and metabolic disorders, but further research is needed to clarify the nature and direction of this relationship.

The impacts of autoprobiotics on the metabolome, both microbial and endogenous, particularly on components of lipid metabolism, is a promising area of research but requires further study [86]. Indeed, our collection of indigenous *Enterococcus* spp. contains strains with different effects. The most effective of them will subsequently be studied genetically and phenotypically, including their antagonistic activity against BFG and *Enterobacteriaceae* species. Their metabolic characteristics and resistance to bile digestive enzymes and hydrochloric acid will also be analyzed. In the future, they may be candidates for the creation of targeted probiotics for the treatment of MetS and associated cardiometabolic disorders.

It should be noted that the limitations of the presented clinical trial are an attribute of the pilot study, and its continuation on a larger scale and expansion in methodological terms are necessary. The small number of patients in each group is related to the fact that only the first (pilot) stage of the study is presented. We focused on patient diaries, which were quite detailed but not tedious, and excluded the loss of patients due to distrust, while remaining committed, which is especially important in the context of a pilot study.

It was important to us that the nutrition medium, which cannot be dispensed when creating an autoprobiotic, does not have a strong therapeutic effect. We acknowledge that this culture medium, containing various nutrients, amino acids, and growth factors, cannot be considered entirely biologically inert. Consequently, it is theoretically possible that the nutritional components of the vehicle could exert minor, independent effects on host metabolism or the native gut microbiota.

Comprehensive characterization of genomic safety of autoprobiotic strains was not performed during this study, as such additional study of a local strain would significantly complicate a clinical trial. However, it is definitely necessary for patients with immune disorders and severe MetS.

The present study did not include whole-genome sequencing of the autoprobiotic *Enterococcus* strains used and was not designed for direct strain-tracking after administration of the PFFP. Therefore, it cannot be completely ruled out that determinants not included in the targeted PCR panel used were present, and the observed changes in the microbiota should not be interpreted as direct evidence of engraftment, long-term persistence, or colonization of the introduced strains. It was not possible in this pilot study to obtain evidence regarding the persistence of indigenous (autoprobiotic) strains. A notable limitation of this study is the absence of strain-level tracking of the administered autologous *Enterococcus* isolates.

While we observed significant shifts in the overall gut microbiota composition following the intervention, we did not confirm the survival, colonization, or persistence of the specific administered strains during and after the treatment period. Therefore, the observed microbial alterations could be a result of either direct engraftment by the autoprobiotics or indirect modulation of the native microbiota. To unequivocally establish the mechanistic link between the intervention and the microbiota shifts, future studies should employ advanced strain-tracking approaches. Techniques such as whole-genome sequencing (WGS) of cultured isolates, strain-specific qPCR, or deep shotgun metagenomic sequencing would allow for precise determination of the survival, persistence, and ecological impact of the administered microorganisms. On the other hand, in the future, it may be possible to identify strains through a genome-wide study, allowing for analysis of their mechanisms of effect. A WGS of autobiotic strains for further possible reuse and biobanking will allow for more widespread use of autobiotics without fear of side effects, including in patients with severe metabolic syndrome and reduced immunity.

## 5. Conclusions

The gut microbiota represents a promising target for personalized microbial therapy of MetS. In patients with MetS, characterized by obesity and disorders of lipid and carbohydrate metabolism, the consumption of indigenous *E. faecium* and *E. hirae* was shown to have potential beneficial effects in the supplementary therapy of MetS, including shorter treatment duration and fewer side effects than invasive medical interventions. This approach should be considered as a strategy to prevent more severe complications, particularly those related to T2DM, cardiovascular diseases, and NAFLD. Based on the literature and our findings, BFG and Senegalimassilia species may serve as potential biomarkers of the response to microbiome-modulating therapies, particularly those involving autoprobiotic Enterococcus strains. 

## Figures and Tables

**Table 1:** Baseline characteristics of participants.

Variables	Ap (n = 26)	Pl (n = 24)	*p*-Value
Age	53.5 (46; 62.25)	53.0 (46; 58.75)	0.4
Sex (M/F)	7/19	8/16	0.622
BW, kg	87.4 (75.4; 99.9)	89 (81.2; 109.7)	0.599
BMI, kg/m^2^	30.6 (28.1; 34.7)	31.5 (30.3; 35.9)	0.641
Waist circumference, cm	102 (94.7; 112.5)	104.5 (94.5; 114.0)	0.46
Hip circumference, cm	114 (106.7; 115.0)	113 (105.2; 120.0)	0.58
Waist-to-Hip Ratio	0.93 (0.86; 0.99)	0.89 (0.86; 0.98)	0.512
Fasting blood sugar, mmol/L	6.2 (5.8; 6.64)	6.2 (5.5; 6.79)	0.456
HbA1c, %	6 (5.75; 6.25)	6.25 (5.8; 6.57)	0.814
Insulin, pmol/L	101.7 (66.5; 151.2)	138.6 (87; 193.3)	0.92
Total cholesterol, mmol/L	5.4 (4.9; 6.2)	5.5 (4.8; 6.6)	0.5
TG, mmol/L	1.3 (1.0; 1.6)	1.7 (1.0; 1.8)	0.784
HDL cholesterol, mmol/L	1.2 (0.9; 1.5)	1.2 (1.0; 1.3)	0.381
VLDL cholesterol, mmol/L	0.6 (0.5; 0.7)	0.7 (0.5; 0.8)	0.775
SBP, mmHg	125 (120; 132)	120 (120; 135)	0.425
DBP, mmHg	80 (76; 89)	80 (79.25; 83)	0.266
CRP, mg/L	2 (0.74; 3.75)	4.25 (2; 6.96)	0.97
Hypertension, %	69	67	0.79
Hypertension + Coronary heart disease, %	31	33	0.642
Statins prescribed, %	25	22	0.504
Antihypertensive drugs, %			
Drinker, %	6	8	0.68
Smoker, %	12	9	0.41

Notes: Data are expressed as median (Me) and 25–75% interquartile range. A chi-square test was performed on categorical variables. BW, Body weight; BMI, Body mass index, SBP, systolic blood pressure; DBP, diastolic blood pressure; HbA1c, glycated hemoglobin; TG, triglycerides; HDL, high-density lipoprotein; VLDL, very low density lipoproteins; CRP, C-reactive protein.

**Table 2:** Anthropometric parameters after consumption of an autoprobiotic or SuproPlus 2640 at different time points.

Parameters	Ap V1	Ap V2	Ap V3	Pl V1	Pl V2	Pl V3	*p* Value
**BW, kg**	87.4 [75.4; 99.9]	86.4 [72.2; 95.5]	85.7 [74.7; 93.5]	89.0 [81.2; 109.7]	89.65 [81.9; 108.2]	89.6 [84.7; 108.5]	Ap V2 vs. Ap V1: *p* = 0.01 Ap V3 vs. Ap V1: *p* = 0.0004 Ap V3 vs. Ap V2: *p* = 0.01
**BMI, kg/m^2^**	30.6 [28.1; 34.7]	29.8 [27.1; 35.2]	29.3 [26.9; 34.4]	31.5 [30.3; 35.9]	31.9 [30.6; 35.8]	32.6 [30.5; 36.7]	Ap V2 vs. Ap V1: *p* = 0.007 Ap V3 vs. Ap V1: *p* = 0.0008 Ap V3 vs. Ap V2: *p* = 0.01
**WC, cm**	102.0 [94.7; 112.5]	99.7 [90.0; 110.25]	97.5 [87.2; 104.5]	104.5 [94.5; 114.0]	101.2 [92.0; 115.5]	100.5 [94.6; 114.0]	Ap V2 vs. Ap V1: *p* = 0.005 Ap V3 vs. Ap V1: *p* < 0.0001 Ap V3 vs. Ap V2: *p* = 0.0003 Pl V2 vs. Pl V1: *p* = 0.02
**HC, cm**	114.0 [106.7; 115.0]	112.0 [106.7; 116.2]	109.5 [104.7; 113.5]	113.0 [105.2; 120.0]	113.5 [102.5; 120.0]	113.0 [104.7; 120.0]	Ap V3 vs. Ap V1: *p* = 0.004 Ap V3 vs. Ap V2: *p* = 0.01
**WHR**	0.93 [0.86; 0.99]	0.89 [0.81; 0.99]	0.89 [0.79; 0.96]	0.89 [0.86; 0.98]	0.88 [0.82; 0.97]	0.88 [0.86; 0.97]	Ap V2 vs. Ap V1: *p* = 0.02 Ap V3 vs. Ap V1: *p* = 0.001

Notes: The experimental (Ap) group and the control (Pl) group consisted of patients with metabolic syndrome who received an autoprobiotic or SuproPlus 2640, respectively. Time points are labeled as Ap V1 or Pl V1 (Day 0), Ap V2 or Pl V2 (Day 49), and Ap V3 or Pl V3 (Day 64). Days 15–34 represent the period of autoprobiotic or SuproPlus 2640 consumption. Results are presented as median (25%; 75%). BW: Body weight; BMI: Body mass index; HC: Hip circumference; WC: Waist circumference; WHR: Waist-to-Hip Ratio (calculated by dividing WC by HC).

**Table 3:** Parameters of the serum lipid profile of patients in the Ap and Pl groups at different time points.

	Ap V1	Ap V2	Ap V3	Pl V1	Pl V2	Pl V3	*p* Value
**Total cholesterol, mmol/L**	5.4 (4.9; 6.2)	5.4 (4.6; 6.0)	4.9 (4.3; 5.7)	5.5 (4.8; 6.6)	5.1 (4.4; 6.4)	5.8 (4.4; 6.5)	Ap V3 vs. Ap V1: *p* = 0.043 Ap V3 vs. Ap V2: *p* = 0.049
**TG, mmol/L**	1.3 (1.0; 1.6)	1.4 (1.2; 2.0)	1.2 (1.0; 1.3)	1.7 (1.0; 1.8)	1.4 (1.2; 1.9)	1.7 (1.3; 2.2)	Ap V3 vs. Ap V2: *p* = 0.001 **Ap V3 vs. Pl V3: *p* = 0.015**
**HDL cholesterol, mmol/L**	1.2 (0.9; 1.5)	1.2 (1.0; 1.5)	1.2 (1.1; 1.7)	1.2 (1.0; 1.3)	1.2 (0.9; 1.4)	1.2 (0.9; 1.4)	Ap V3 vs. Ap V1: *p* = 0.002 Ap V3 vs. Ap V2: *p* = 0.008
**VLDL cholesterol, mmol/L**	0.6 (0.5; 0.7)	0.6 (0.5; 0.9)	0.6 (0.5; 0.7)	0.8 (0.5; 0.8)	0.6 (0.5; 0.9)	0.8 (0.6; 1.0)	Ap V3 vs. Ap V1: *p* = 0.033 Ap V3 vs. Ap V2: *p* = 0.027

Notes: The experimental (Ap) group and the control (Pl) group consisted of patients with metabolic syndrome who received an autoprobiotic or SuproPlus 2640, respectively. Time points are labeled as Ap V1 or Pl V1 (Day 0), Ap V2 or Pl V2 (Day 49), and Ap V3 or Pl V3 (Day 64). Days 15–34 represent the period of autoprobiotic or SuproPlus 2640 consumption. Results are presented as median (25%; 75%). HDL: High-density lipoproteins; TG: Triglycerides; VLDL: Very low-density lipoproteins.

## Data Availability

The original contributions presented in this study are included in the article/supplementary material. Further inquiries can be directed to the corresponding authors.

## Supplementary Materials

The following supporting information can be downloaded at: https://www.mdpi.com/article/10.3390/nu18142324/s1, (1) Recommendations for physical activity and diet for all patients with MetS2 and its references [87,88] are cited in the supplementary materials; (2) Figures (Figure S1 Participant flow diagram. Figure S2. Alpha-diversity indices by study group and visit. Figure S3A. Principal coordinates analysis based on Bray-Curtis distance across all visits. Notes: Point color red (Ap) and violet (Pl) indicates treatment group. Figure S3B. Principal coordinates analysis based on Bray-Curtis distance at baseline (V1) only. Figure S3C. Principal coordinates analysis based on Jaccard distance across all visits. Figure S3D. Principal coordinates analysis based on Jaccard distance at baseline (V1) only. Figure S4. Spearman correlation matrix of ASV/taxonomic features); Tables (Table S1. Availability of fecal samples by group by group and visit, Table S2. Availability of blood serum samples by group and visit. Table S3. Autoprobiotic strains characteristics, Table S4. Genes which were detected for predefined determinants (for PCR/electrophoretic),Table S5. ASV/feature table summary. Table S6. Main interaction test group × visit for alpha-diversity indicators. Table S7. Estimated intergroup differences Ap − Pl within visits for key alpha-diversity indicators. Table S8. Difference-in-differences contrasts for key alpha-diversity metrics/Table S9. Exploratory responder/non-responder counts пo Shannon V3−V1. Table S10. Fisher exact test for responders shares. Table S11. PERMANOVA: group × visit interaction and initial comparability of Ap and Pl on V1. Table S12. PERMDISP: Checking the heterogeneity of variances for beta-diversity. Table S13. Mixed models for distance from own baseline by beta-diversity metrics. Table S14. Intergroup differences in distance-from-baseline at follow-up).

## Abbreviations

BFG	*Bacteroides fragilis* group
BMI	body mass index
BW	body weight
CRP	C-reactive protein
DBP	diastolic blood pressure
HbA1c	glycated hemoglobin
HDL	high-density lipoprotein
HC	hip circumference
MetS	metabolic syndrome
PFFP	personalized functional food product
SBP	systolic blood pressure
T2DM	type 2 diabetes mellitus
TG	triglyceride
VLDL	very low-density lipoprotein
WC	waist circumference
WHR	waist-to-hip ratio
ISRCTN	International Traditional Clinical Trials Registry
NAFLD	non-alcoholic fatty liver disease
WGS	whole-genome sequencing
