# Supplementary material for: Autoprobiotic Supplements Attenuate Obesity and Improve Gut Microbiota, Carbohydrate, and Lipid Metabolism in Patients with Metabolic Syndrome: A Pilot Trial
Source: Nutrients. 2026 Jul 16;18(14):2324. doi: 10.3390/nu18142324 (PMC13414959; doi:10.3390/nu18142324)
Supplement: Supplementary file 1 [file nutrients-18-02324-s001.zip › nutrients-4252583-supplementary.pdf]

Supplementary Materials:

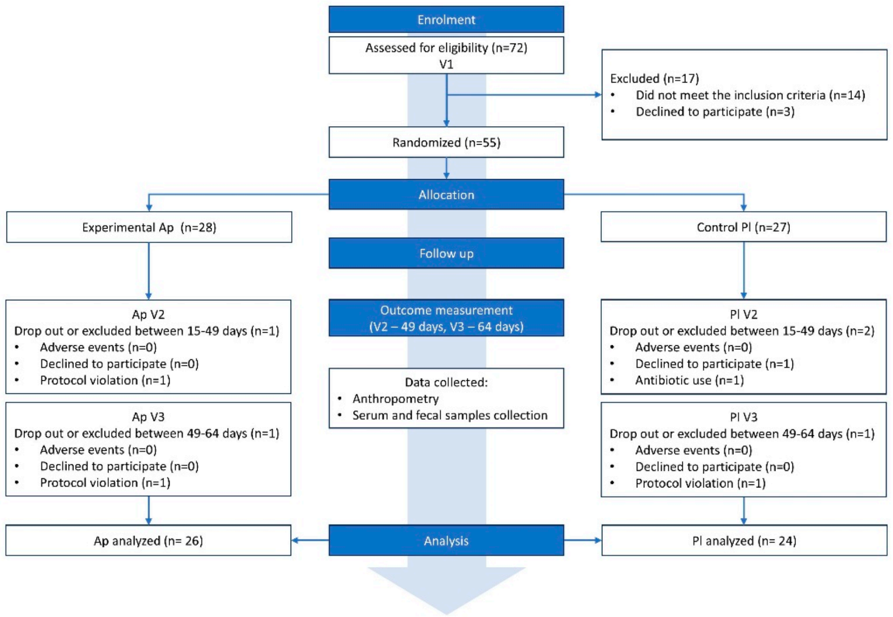

Figure S1. Participant flow diagram.

RECOMMENDATIONS FOR PHYSICAL ACTIVITY AND DIET FOR ALL PATIENTS with MetS

All patients were given physical activity recommendations in accordance with WHO guidelines (available in Russian https://www.who.int/ru/news-room/fact-sheets/detail/physical-activity). Recommendations were individualized based on age, baseline physical activity (using the GPAQ questionnaire in Russian), and physical activity tolerance assessed by anamnesis of physical activity (for all patients), exercise stress testing (for some patients who had previously completed it), or the DASI scale (for some patients, if it had already been used or the study physician deemed its use appropriate). A specific, precise assessment of exercise tolerance was not conducted, as this was not the primary objective of this study.

Dietary recommendations for all patients were consistent with current National Guidelines for Lipid Metabolism Disorders [87] and Diabetes Mellitus Type 2 [88], and other recommendations.

Specifically, to reduce the risk of cardiovascular disease, the Mediterranean diet and its analogues were recommended, along with replacing saturated fats with unsaturated fats and limiting salt intake. Consuming more fiber-rich plant foods, including whole grains, fruits, vegetables, legumes and nuts, and fish, especially fatty ones, at least once a week, and limiting processed meats were recommended. Limiting simple carbohydrate intake, particularly sugar-sweetened beverages, to a maximum of 10% of energy intake was recommended. If the study physician deemed the patient already following a healthy diet, they recommended maintaining their eating habits for the duration of the study.

Table S1. Availability of fecal samples by group by group and visit.

Visits /Samples quantity	V1	V2	V3
Ap	26	26	26
Pl	24	24	24

Table S2. Availability of blood serum samples by group and visit.

Visits /Samples quantity	V1	V2	V3
Ap	26	25	25
Pl	24	24	24

Table S3. Autoprobiotic strains characteristics.

Patient_ id	Strain_id	Species	Method	amr_gene	Virulence _ gene	Detected	Safety_ comment
1	Efaecium_001	Enterococcus faecium	PCR / targeted molecular assay; MALDI-TOF for species identification	example_gene	example_ gene*	No	No clinically relevant determinant detected
1	Ehirae_001	Enterococcus hirae	PCR / targeted molecular assay; MALDI-TOF for species identification	example_gene	example_ gene	No	No clinically relevant determinant detected

Note: The *Enterococcus* indigenous strains used for the preparation of the autoprobiotic product were identified to the species level using PCR and MALDI-TOF MS. Before inclusion in the production of the product, the isolates underwent targeted molecular safety screening using PCR/electrophoretic detection for predefined determinants, including pathogenicity-associated genes and vancomycin-resistance genes, as listed in Supplementary Table S14. Only isolates classified as *E. faecium* or *E. hirae* and not containing determinants associated with pathogenicity or vancomycin and erythromycin resistance identified within this panel were used to prepare the preparation. Whole-genome sequencing was not performed.

Table S4. Genes which were detected for predefined determinants (for PCR/electrophoretic),

Function	Factor	Genes
**Adhesion and colonization**	Capsule	*cps*
	Esp adhesin	*esp*
	Asa adhesin	*asa1*
	EfaA adhesin	*efaA*
	Aggregation factor	*agg*
	Collagen receptor	*ace*
**Penetration, colonization, tissue damage**	Gelatinase	*gelE*
	Serine protease	*spr*
	Fsr regulator	*fsr*
	Hyaluronidase	*hylEfm*
**Resistance to antibiotics and**	Antibiotic resistance	*vanA,B,C, ermB*

Microbiome 16S rRNA analysis

Table S5. ASV/feature table summary.

Number of ASV/features	4972
Samples with non-zero ASV profile	121
Non-zero ASV/features per sample, median [Q1; Q3]	78 [58; 136]
ASV/features after prevalence filtering ≥ 10%	184

Alpha-diversity

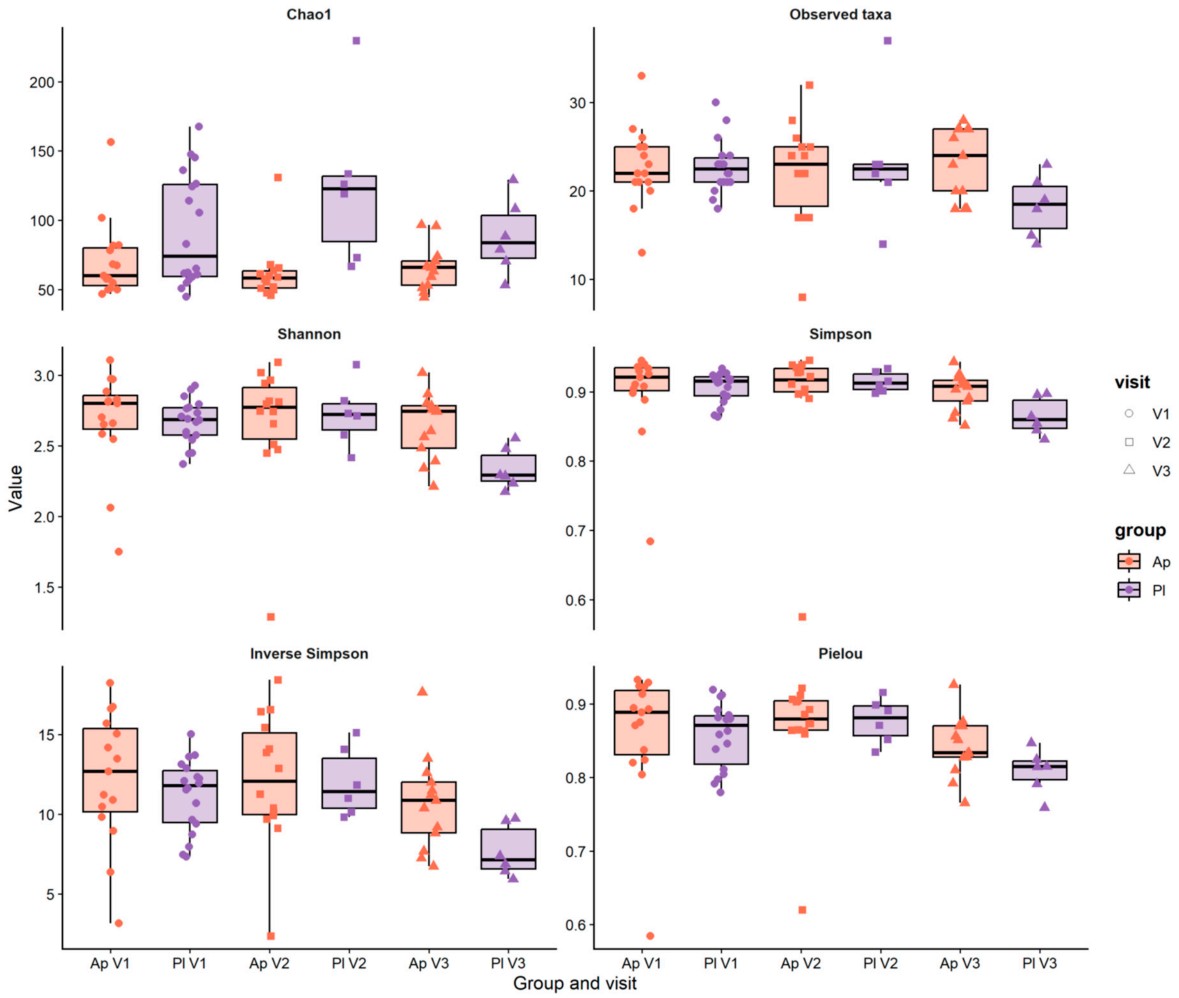

Figure S2. Alpha-diversity indices by study group and visit.

Table S6. Main interaction test group × visit for alpha-diversity indicators.

metric	term	Sum Sq	Mean Sq	NumDF	DenDF	F value	Pr(>F)
chao1	group:visit	3915	1958	2	39.05	4.262	0.0212
observed_taxa	group:visit	101.2	50.59	2	48.65	3.541	0.0367
shannon	group:visit	0.2297	0.1148	2	41.65	2.797	0.0725
simpson	group:visit	0.0041	0.0021	2	38.06	1.431	0.2516
inverse_simpson	group:visit	15.79	7.895	2	46.3	0.9578	0.3912
pielou	group:visit	0.0021	0.0011	2	30.64	0.505	0.6084

Table S7. Estimated intergroup differences Ap – Pl within visits for key alpha-diversity indicators.

metric	contrast_type	contrast	visit	estimate	SE	df	lower.CL	upper.CL
chao1	Ap minus Pl within visit	Ap - Pl	V1	-21.4	11.57	55.41	-44.58	1.783
chao1	Ap minus Pl within visit	Ap - Pl	V2	-44.91	14.99	75.67	-74.77	-15.06
chao1	Ap minus Pl within visit	Ap - Pl	V3	0.7806	15.1	76.12	-29.3	30.86
observed_taxa	Ap minus Pl within visit	Ap - Pl	V1	-0.0792	1.621	70.84	-3.311	3.152
observed_taxa	Ap minus Pl within visit	Ap - Pl	V2	-1.474	2.241	78.3	-5.936	2.988
observed_taxa	Ap minus Pl within visit	Ap - Pl	V3	5.227	2.265	78.41	0.7177	9.736
shannon	Ap minus Pl within visit	Ap - Pl	V1	0.0018	0.1057	57.48	-0.2099	0.2134
shannon	Ap minus Pl within visit	Ap - Pl	V2	-0.1156	0.1384	76.43	-0.3912	0.1599
shannon	Ap minus Pl within visit	Ap - Pl	V3	0.2271	0.1395	76.82	-0.0507	0.5049

Table S8. Difference-in-differences contrasts for key alpha-diversity metrics

metric	contrast_type	contrast	estimate	SE	df	lower.CL		upper.CL
chao1	Difference-in-differences	Difference-in-differences: Ap vs Pl, V2 minus V1	-23.52	14.69	50.39	-53.02		5.99
chao1	Difference-in-differences	Difference-in-differences: Ap vs Pl, V3 minus V1	22.18	14.92	51.09	-7.784		52.14
observed_taxa	Difference-in-differences	Difference-in-differences: Ap vs Pl, V2 minus V1	-1.395	2.498	54.93	-6.4		3.61
observed_taxa	Difference-in-differences	Difference-in-differences: Ap vs Pl, V3 minus V1	5.306	2.53	56.1	0.2384		10.37
shannon	Difference-in-differences	Difference-in-differences: Ap vs Pl, V2 minus V1	-0.1174	0.1382	50.77	-0.395		0.1601
shannon	Difference-in-differences	Difference-in-differences: Ap vs Pl, V3 minus V1	0.2253	0.1404	51.52	-0.0564		0.5071

Table S9. Exploratory responder/non-responder counts по Shannon V3–V1.

group	responder_status	n	group_total	percent
Ap	Non-responder	7	12	58.33
Ap	Responder	5	12	41.67
Pl	Non-responder	6	6	100

Table S10. Fisher exact test for responders shares.

comparison	odds_ratio	conf_low	conf_high	p_value
Responder proportion: shannon V3 vs V1	0	0	1.95	0.1141

<u>**Beta- diversity (by PERMANOVA)**</u>

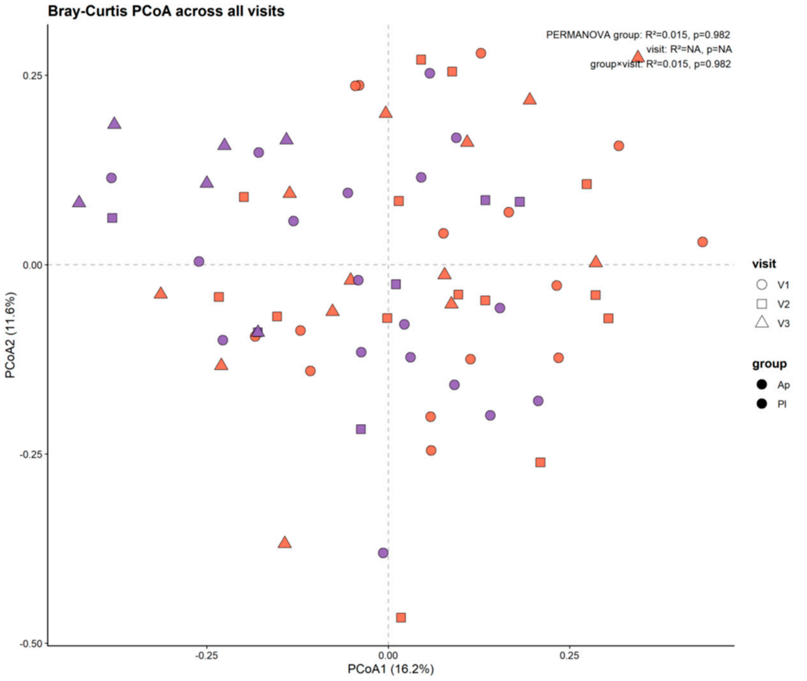

Figure S3A. Principal coordinates analysis based on Bray-Curtis distance across all visits. Notes: Point color red (Ap) and violet (Pl) indicates treatment group.

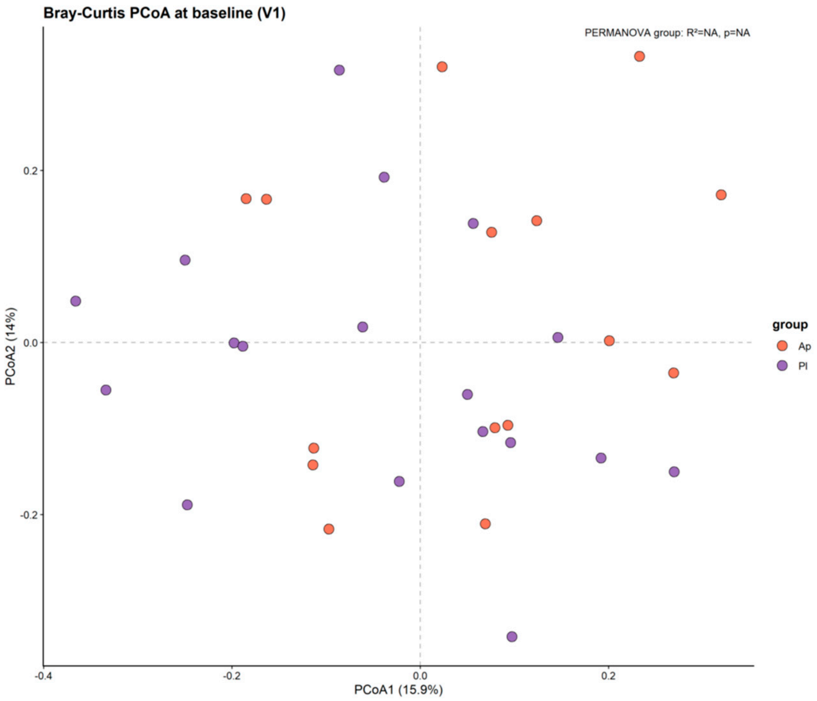

Figure S3B. Principal coordinates analysis based on Bray-Curtis distance at baseline (V1) only. Notes: Point color red (Ap) and violet (Pl) indicates treatment group.

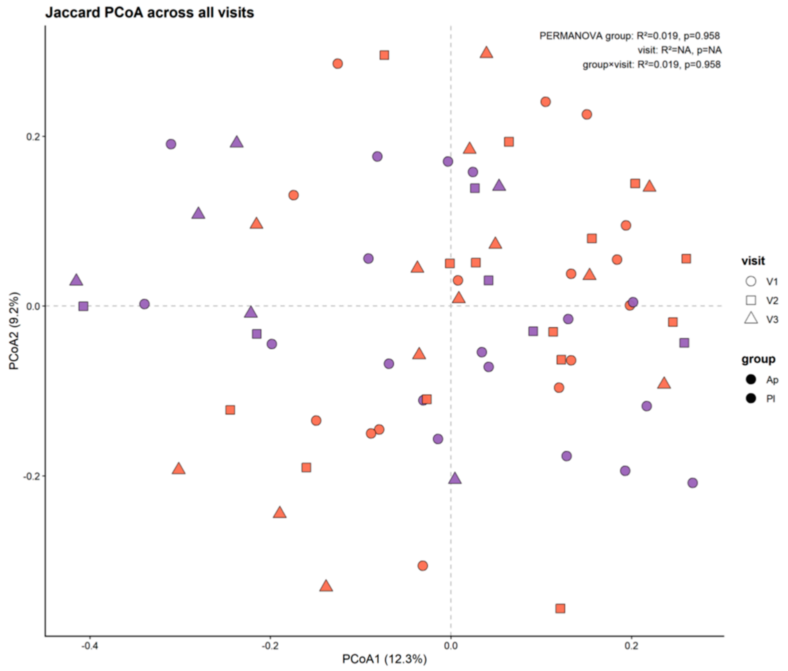

Figure S3C. Principal coordinates analysis based on Jaccard distance across all visits.

Note: Point color red (Ap) and violet (Pl) indicates treatment group..

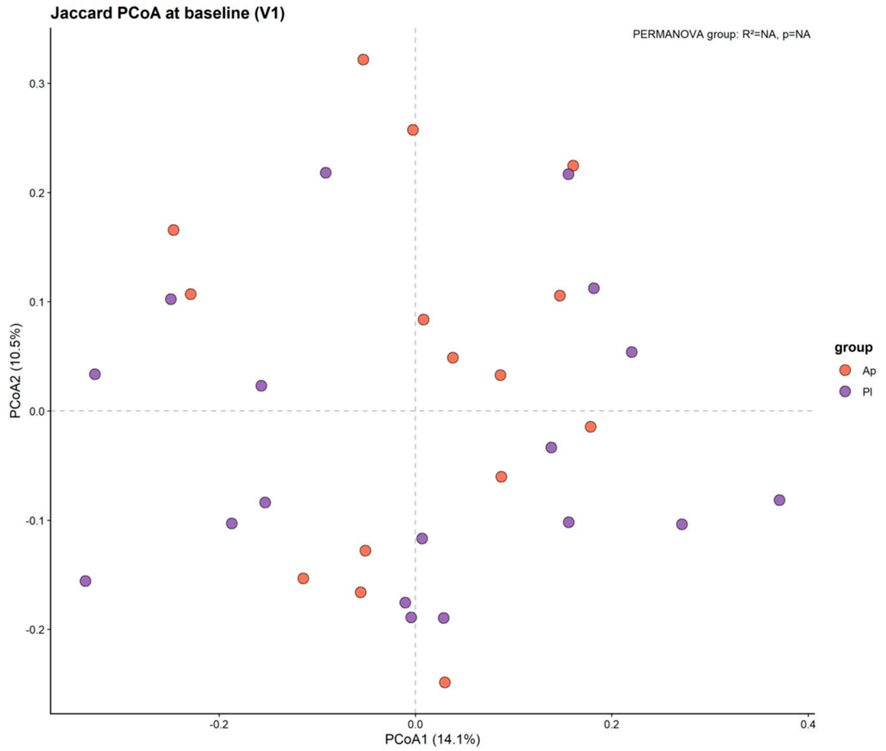

Figure S3D. Principal coordinates analysis based on Jaccard distance at baseline (V1) only.
Notes: Point color (Ap)red and violet (Pl) indicates treatment group.

PERMANOVA results for beta-diversity distances. Bray–Curtis: $R^2 = 0.134$, $p = 0.001$; Jaccard: $R^2 = 0.108$, $p = 0.020$; Aitchison: $R^2 = 0.108$, $p = 0.024$.

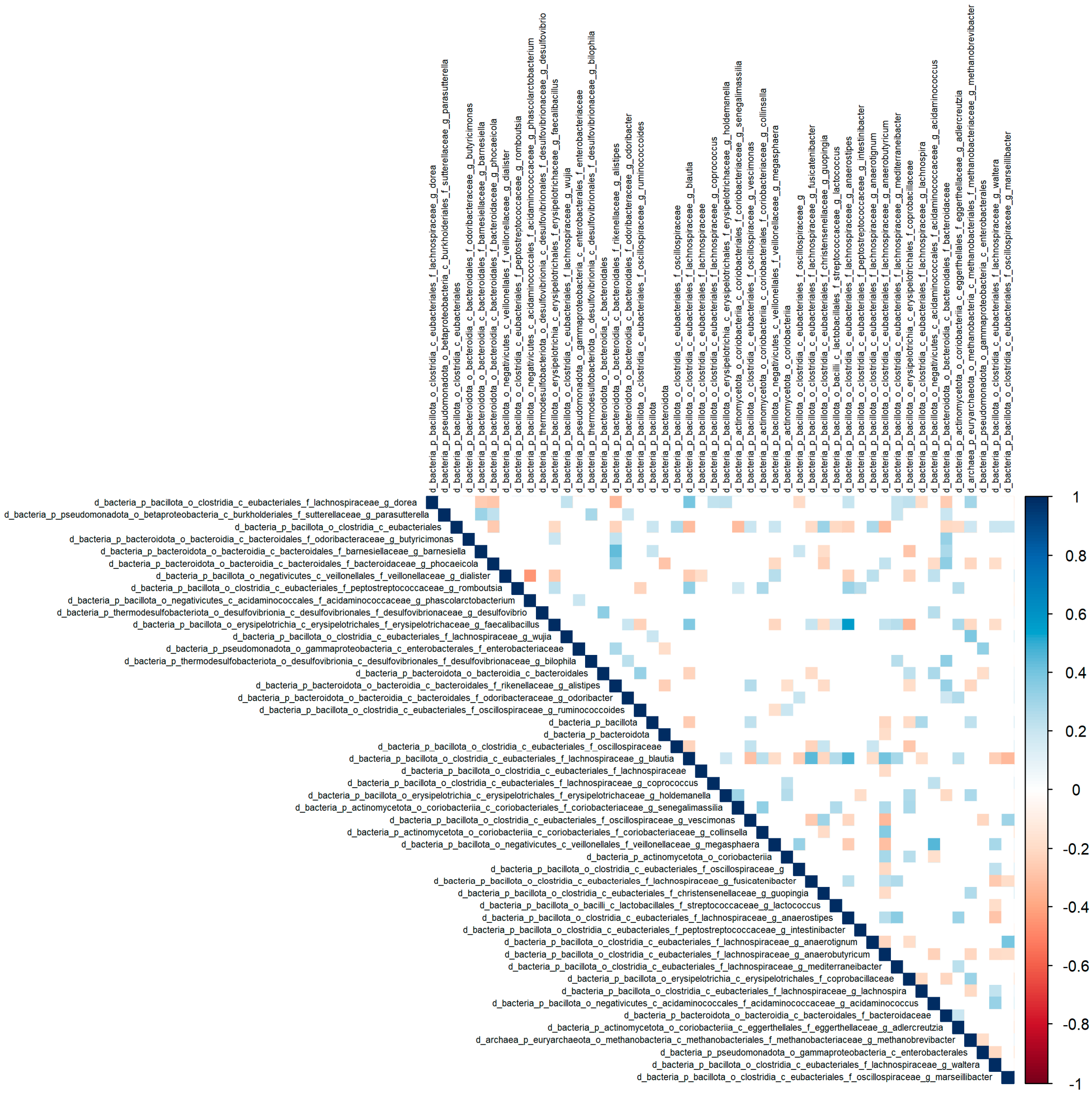

Figure S4. Spearman correlation matrix of ASV/taxonomic features.

Table S11. PERMANOVA: group × visit interaction and initial comparability of Ap and Pl on V1.

metric	analysis	Df	SumOfSqs	R2	F	Pr(>F)	term
Bray-Curtis	All visits: group * visit	2	0.2151	0.0151	0.5432	0.9822	group:visit
Bray-Curtis	Baseline V1: Ap vs Pl	1	0.2253	0.0365	1.173	0.2637	Model
Jaccard	All visits: group * visit	2	0.2943	0.0188	0.6672	0.9578	group:visit
Jaccard	Baseline V1: Ap vs Pl	1	0.2205	0.0328	1.05	0.3971	Model
Aitchison	All visits: group * visit	2	156.8	0.018	0.639	0.9896	group:visit
Aitchison	Baseline V1: Ap vs Pl	1	121.7	0.0313	1.003	0.4392	Model

Table S12. PERMDISP: Checking the heterogeneity of variances for beta-diversity.

metric	analysis	Df	Sum Sq	Mean Sq	F value	Pr(>F)	term
Bray-Curtis	All visits: dispersion by group_visit	5	0.043	0.0086	1.464	0.2137	Groups
Bray-Curtis	Baseline V1: dispersion by group	1	0.0054	0.0054	1.12	0.2982	Groups
Jaccard	All visits: dispersion by group_visit	5	0.0067	0.0013	0.3819	0.8595	Groups
Jaccard	Baseline V1: dispersion by group	1	0.0002	0.0002	0.1005	0.7534	Groups
Aitchison	All visits: dispersion by group_visit	5	15.02	3.003	2.119	0.074	Groups
Aitchison	Baseline V1: dispersion by group	1	0.8158	0.8158	0.8627	0.3602	Groups

Table S13. Mixed models for distance from own baseline by beta-diversity metrics.

metric	term	Sum Sq	Mean Sq	NumDF	DenDF	F value	Pr(>F)
Bray-Curtis	group	0.0076	0.0076	1	38	0.6484	0.4257
Bray-Curtis	visit	0.0137	0.0137	1	38	1.161	0.288
Bray-Curtis	group:visit	0.01	0.01	1	38	0.8451	0.3637
Jaccard	group	0.0202	0.0202	1	38	2.76	0.1049
Jaccard	visit	0.0105	0.0105	1	38	1.431	0.239
Jaccard	group:visit	0.003	0.003	1	38	0.4117	0.5249
Aitchison	group	0.5249	0.5249	1	38	0.1933	0.6627
Aitchison	visit	5.352	5.352	1	38	1.971	0.1685
Aitchison	group:visit	0.0051	0.0051	1	38	0.0019	0.9656

Table S14. Intergroup differences in distance-from-baseline at follow-up visits.

metric	contrast_type	contrast	visit	estimate	SE	df	lower.CL	upper.CL
Bray-Curtis	Ap minus Pl within follow-up visit	Ap - Pl	V2	0.0654	0.0566	42.47	-0.0488	0.1797
Bray-Curtis	Ap minus Pl within follow-up visit	Ap - Pl	V3	-0.0043	0.0582	42.47	-0.1217	0.1131
Jaccard	Ap minus Pl within follow-up visit	Ap - Pl	V2	-0.0304	0.0446	42.47	-0.1204	0.0595
Jaccard	Ap minus Pl within follow-up visit	Ap - Pl	V3	-0.0688	0.0458	42.47	-0.1612	0.0236
Aitchison	Ap minus Pl within follow-up visit	Ap - Pl	V2	0.2781	0.8594	42.47	-1.456	2.012
Aitchison	Ap minus Pl within follow-up visit	Ap - Pl	V3	0.2281	0.8831	42.47	-1.553	2.01
